# Supplementary material for: Molecular basis and cellular functions of vinculin-actin directional catch bonding
Source: Nat Commun. 2023 Dec 14;14:8300. doi: 10.1038/s41467-023-43779-x (PMC10721916; doi:10.1038/s41467-023-43779-x)
Supplement: Supplementary file 1 — Supplemental information [file 41467_2023_43779_MOESM1_ESM.pdf]

Supplementary Materials for

Molecular Basis and Cellular Functions of Vinculin-Actin Directional Catch Bonding

Venkat R. Chirasani,[1]† Mohammad Ashhar I. Khan,[1]† Juilee N. Malavade,[2]† Nikolay V. Dokholyan,[3,4,5] Brenton D. Hoffman,[2,6]* Sharon L. Campbell[1,7]*

†These authors contributed equally to this work.

*Corresponding authors. Email: brenton.hoffman@duke.edu (B.D.H.); campbesl@med.unc.edu (S.L.C.)

This file includes:

Supplementary Figures 1 to 20
Supplementary Tables 1 to 22

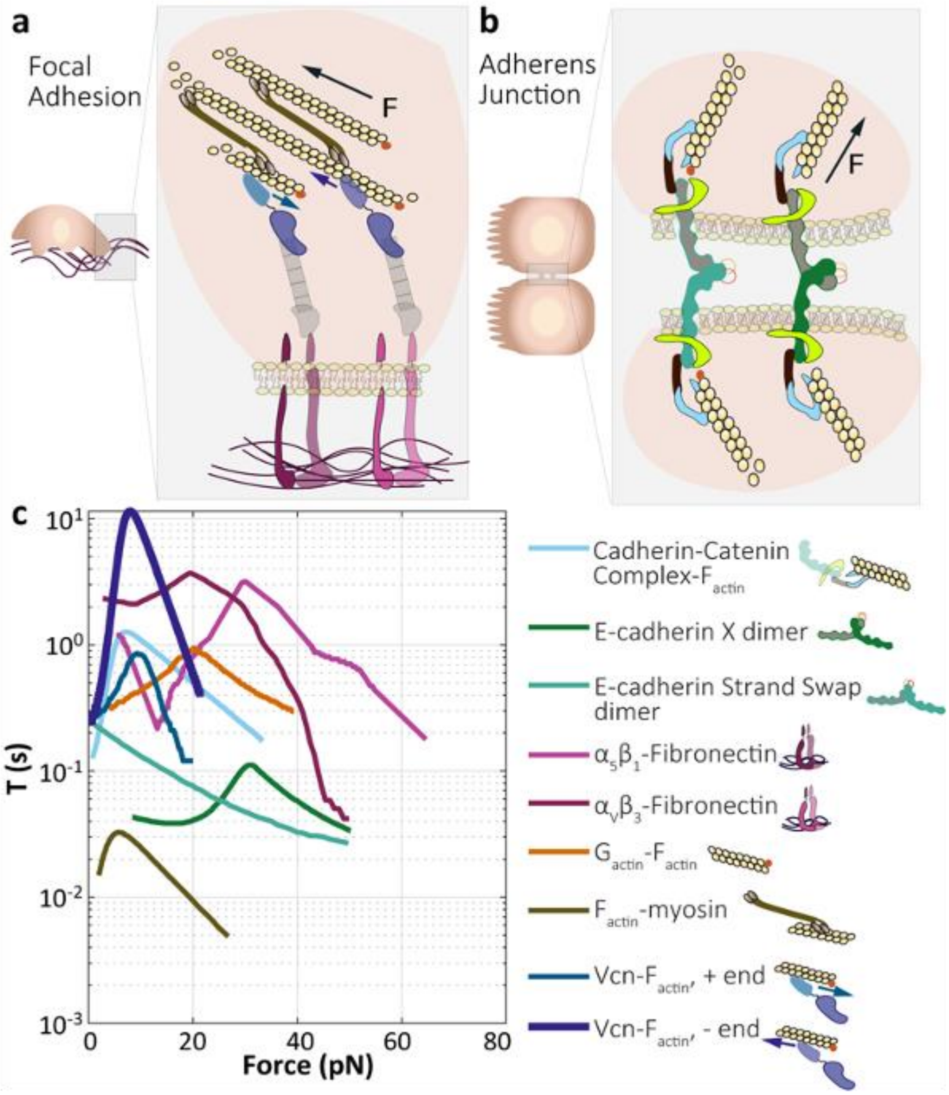

Supplementary Figure 1. Lifetimes of single interface protein-protein interactions at FAs and AJs.

Schematics of force-sensitive cellular adhesion structures **a,** linking the cell to the extracellular matrix or **b,** to other cells. **c,** Bond lifetimes for single interface protein-protein interactions measured via single molecule force spectroscopy studies (SMFS) at FAs and AJs.

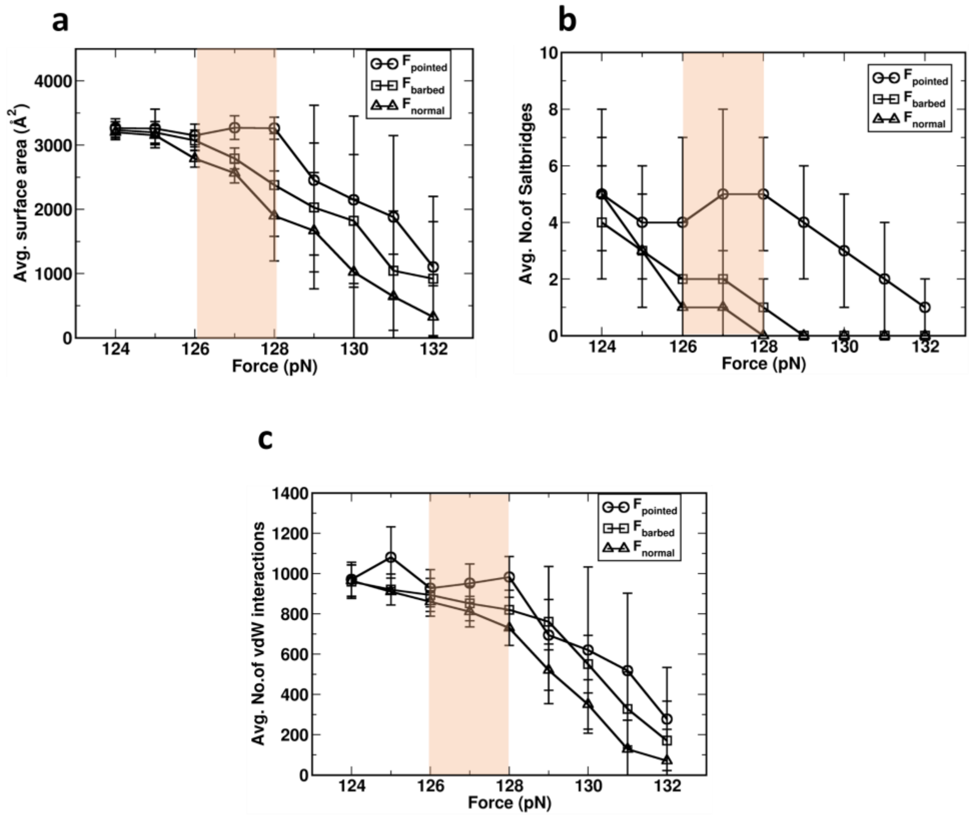

Supplementary Figure 2. Evaluation of contact area, salt-bridges, and van der Waals (vdW) interactions between Vt and F-actin.

Quantification of the **a,** contact area, **b,** average number of salt-bridge interactions, and **c,** average number of vdW interactions between Vt and F-actin from constant-force pulling DMD simulations in $F_{pointed}$, F_{barbed}, and F_{normal} directions. A cut-off distance of 3.5 Å and 5 Å was employed to evaluate salt-bridge and vdW interactions, respectively, using VMD. The sidechains of charged residues exhibit dynamic behavior and undergo transient interactions, resulting in higher uncertainty and variability. The *gmx sasa* tool was used to plot contact area versus pulling force. Pulling force range in the range of 126 – 128 pN is highlighted to indicate the DAFS interaction force regime. Source data are provided as a Source Data file.

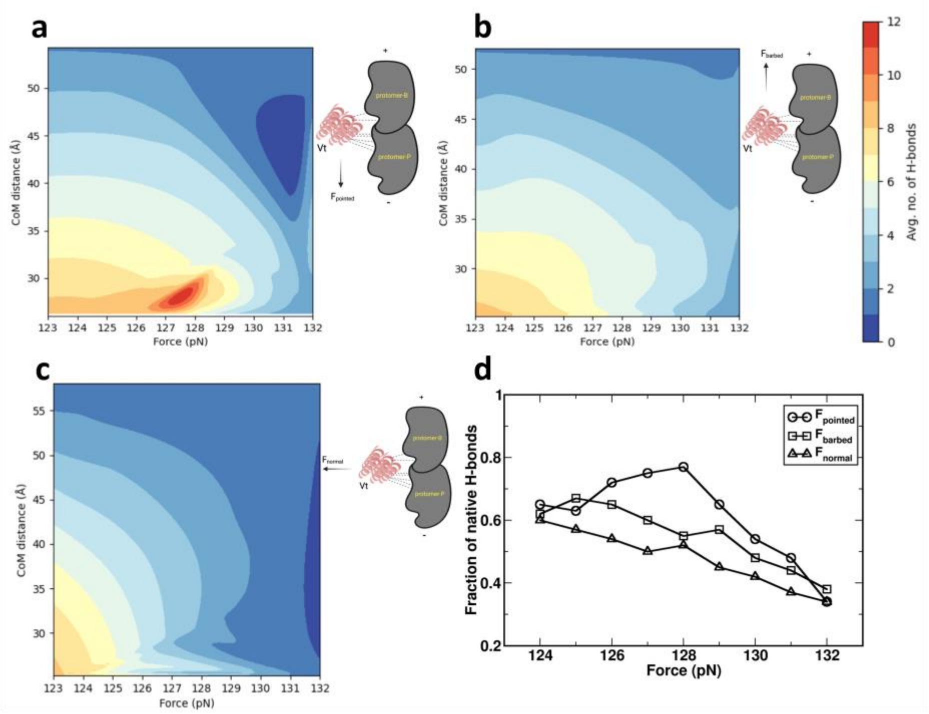

Supplementary Figure 3. H-bond density plots and fraction of native H-bonds from DMD pulling simulations in three pulling directions.

Analysis of H-bonds between Vt and F-actin as a function of different pulling directions. Density maps of the average number of H-bonds as a function of force and Vt:F-actin center of mass (CoM) distance are shown in Panels **a-c**, highlighting strong and weak interactions between the two molecules. The engagement of DAFS interactions results in a higher number of H-bonds between Vt and F-actin in the $F_{pointed}$ direction. Panel **d** shows the fraction of native H-bonds preserved between F-actin and Vt in the various pulling directions and loading conditions, as calculated by the ratio of the number of H-bonds present in the pulled state to the number of H-bonds in the unloaded state (PDB: 3JBI). H-bonds with occupancy > 10% were employed for evaluation of the fraction of native H-bonds.

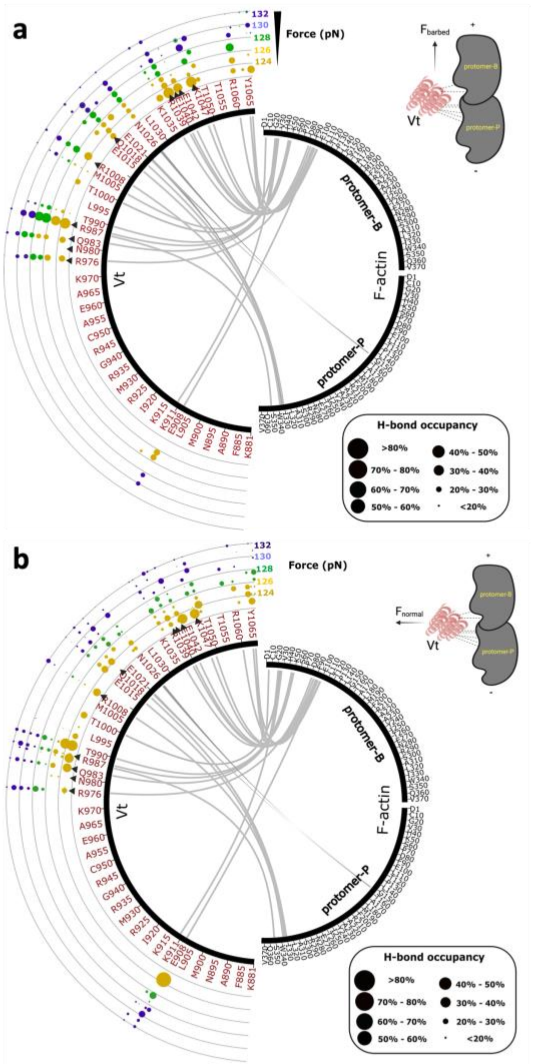

Supplementary Figure 4. Occupancy percentages of inter-molecular H-bonds between Vt and F-actin.

To investigate Vcn:F-actin engagement in the **a,** F_{barbed} and **b,** F_{normal} directions, circular plots of Vt (red) and F-actin (black) interactions were generated. The occupancy percentages of intermolecular H-bond interactions between F-actin and Vt obtained from constant-force pulling DMD simulations were used to create the circular plots. Each dot on the scatter plot represents the presence of an H-bond interaction at different pulling forces, with the color indicating the magnitude of the applied force. The size of each dot corresponds to the occupancy percentage of the respective H-bond during the pulling simulation at a specific force. Native H-bonds observed in the unloaded state based on the cryo-EM structure of the Vt:F-actin complex (PDB: 3JBI) are depicted as black triangles on the heatmap with the intermolecular H-bonds visualized as grey ribbons, providing a representation of the spatial arrangement of these interactions.

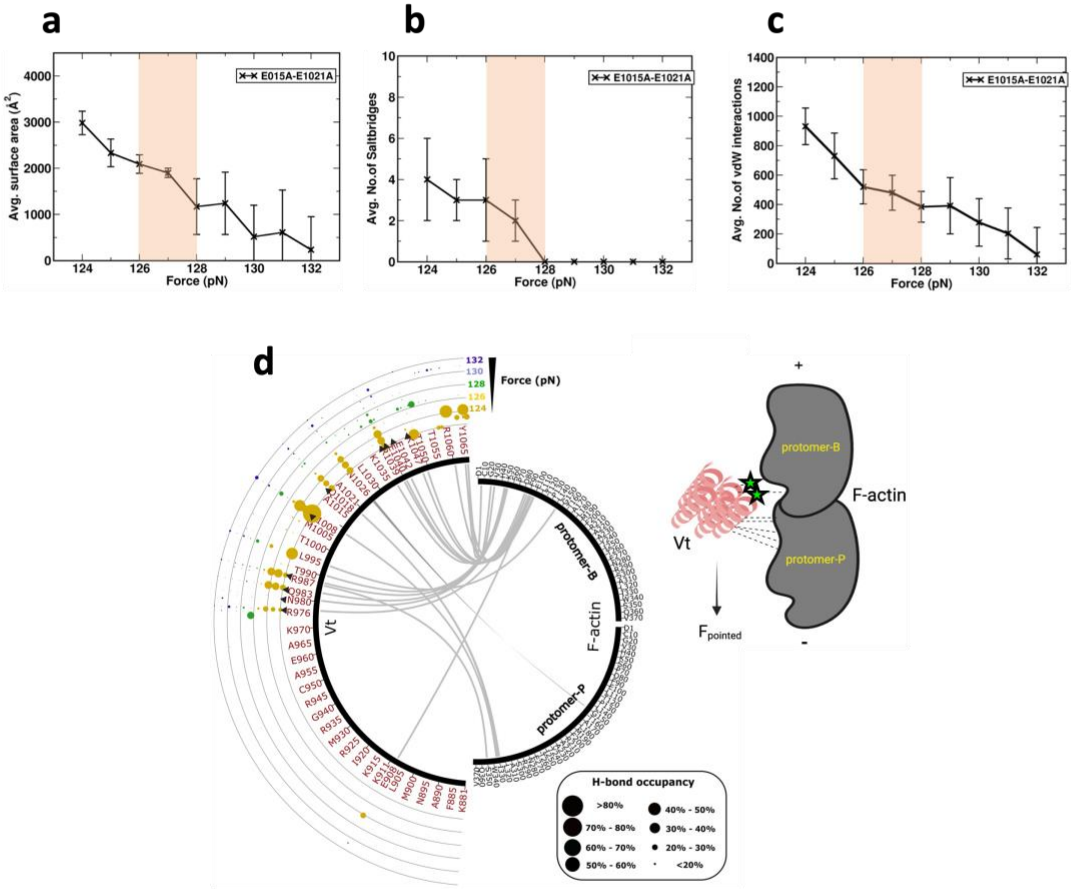

Supplementary Figure 5. DMD simulations predict that the Vt E1015A-E1021A variant abolishes most DAFS interactions.

To validate the presence of DAFS interactions, a Vt variant (E1015A-E1021A) was introduced into the Vt:F-actin complex, and its engagement with F-actin in the $F_{pointed}$ direction was investigated through constant-force pulling DMD simulations. Quantification of the **a,** contact area, **b,** average number of salt-bridge interactions, and **c,** average number of vdW interactions between Vt_E1015A-E1021A and F-actin in the $F_{pointed}$ direction. A cut-off distance of 3.5 Å and 5 Å was employed to evaluate salt-bridge and vdW interactions, respectively, using VMD. The *gmx sasa* tool was used to plot contact area versus pulling force. Pulling force in the range of 126 – 128 pN is highlighted to indicate the DAFS interaction force regime. Source data are provided as a Source Data file. **d,** Circular plot illustrating intermolecular H-bond occupancies between the Vt_E1015A-E1021A variant (red) and F-actin (black) at different pulling forces in the $F_{pointed}$ direction is shown. Each dot on the plot represents a H-bond interaction, with the color of the dot indicating the magnitude of the applied force and the size of the dot corresponds to the H-bond occupancy percentage. Native H-bond interactions observed in the unloaded state (based on the cryo-EM structure of the Vt:F-actin complex (PDB: 3JBI)), are depicted as black triangles on the heatmap. The H-bond interactions are visualized as grey ribbons, providing a representation of the spatial arrangement of these interactions.

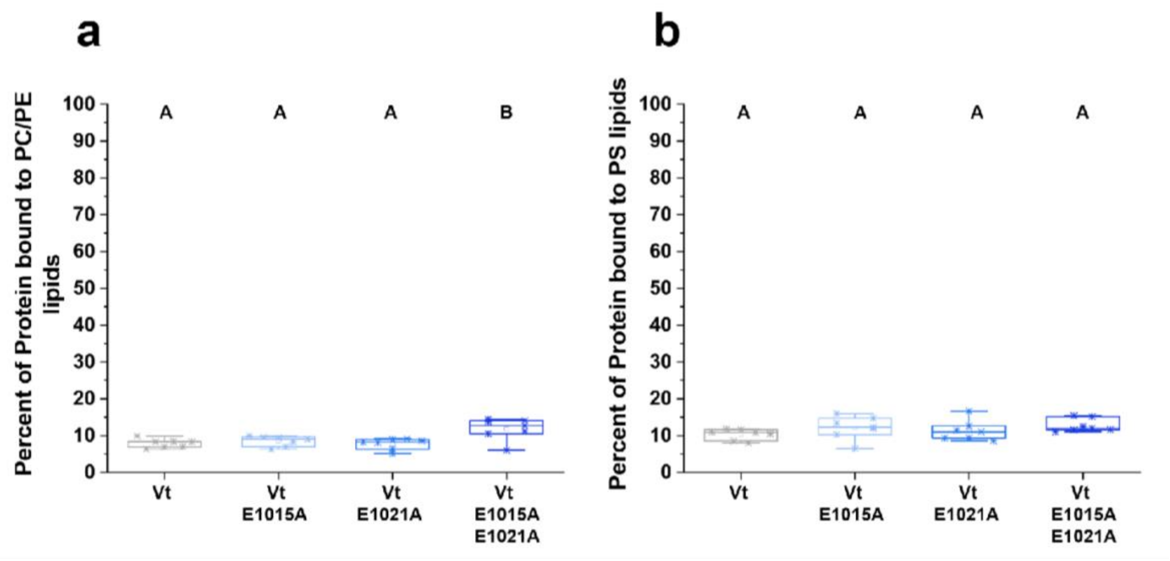

Supplementary Figure 6. DAFS variants retain the Vt:lipid interactions.

Lipid co-sedimentation assay comparing association of WT Vt and DAFS Vt variants with large unilamellar vesicles (LUVs). The DAFS Vt variants exhibit similar lipid binding for **a,** neutral and **b,** anionic phospholipids relative to WT Vt. Box-whisker plots are based on n=7 independent measurements from N=2 preparations. Box depicts the median as the center value, the 25th percentile as the lower bound, and the 75th percentile as the upper bound. Whiskers extend 1.5 times the interquartile region (IQR) from the bottom and top of box, or to the minimum and maximum of the data if the data does not extend to the whiskers. Values outside the whiskers are plotted as individual points. One-way ANOVA and Tukey's HSD Test used for statistical analysis. Different letters denote significant differences at $p<0.05$. See Supplementary Tables 10-11 for a detailed listing of p-values. Source data are provided as a Source Data file.

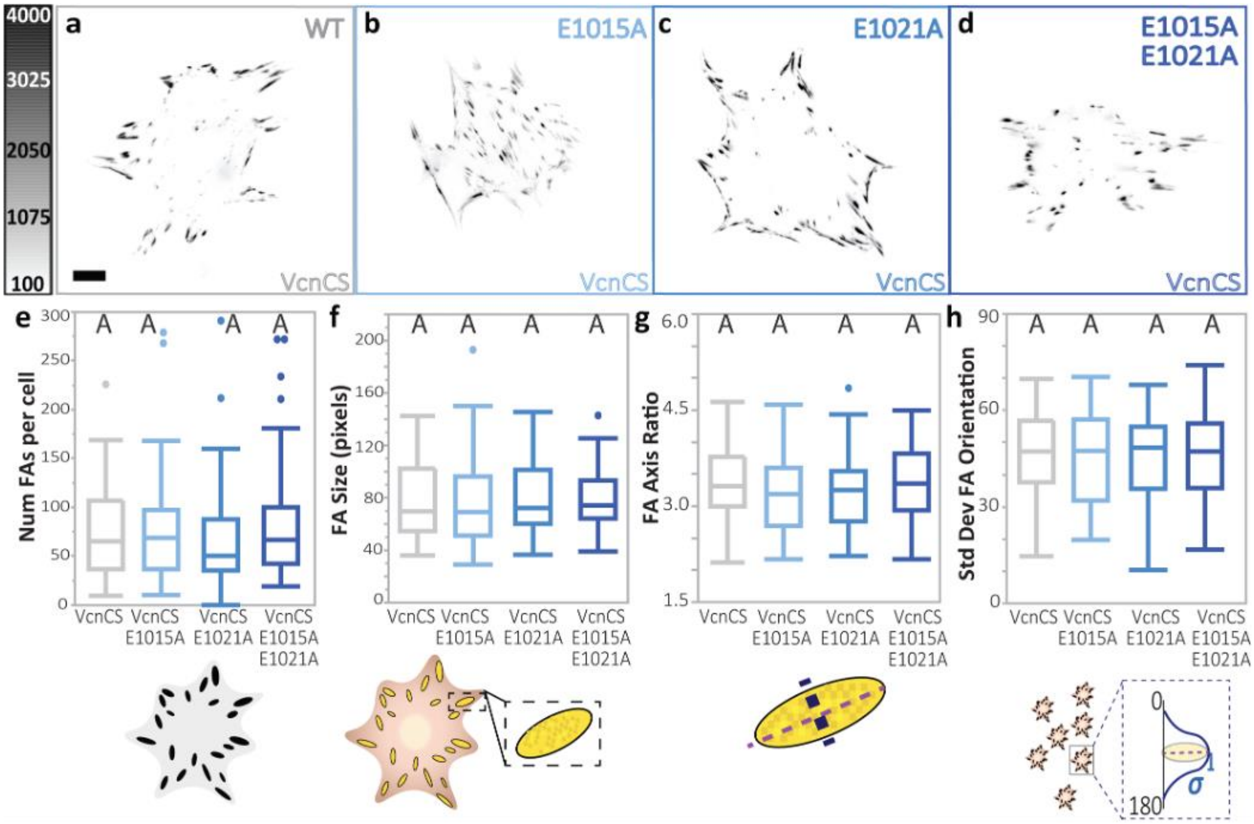

Supplementary Figure 7. VcnCS DAFS variants do not display altered FA characteristics. **a-d,** Representative images of Vcn$^{-/-}$ MEFs expressing WT VcnCS or VcnCS DAFS variants. WT VcnCS is shown in gray and variants of VcnCS are shown in shades of blue. Scale bar: 10 µm. Quantification of FA morphometric characteristics are shown including **e,** FA number, **f,** FA area, **g,** FA axis ratio, and **h,** standard deviation of FA orientation. Plots shown for WT VcnCS and VcnCS DAFS variants (*n*= 37, 41, 47, 53 cells, respectively, collected over N=3 independent experiments). Box depicts the median as the center value, the 25th percentile as the lower bound, and the 75th percentile as the upper bound. Whiskers extend 1.5 times the IQR from the bottom and top of box, or to the minimum and maximum of the data if the data does not extend to the whiskers. Values outside the whiskers are plotted as individual points. One-way ANOVA paired with a Steel-Dwass test (e, f, g) or Tukey's HSD test h, was conducted across all VcnVenus, VcnCS and VcnTS constructs used for statistical analysis. Differing letters denote significant difference at p<0.05. See Supplementary Tables 4-7 for a detailed listing of p-values. Source data are provided as a Source Data file.

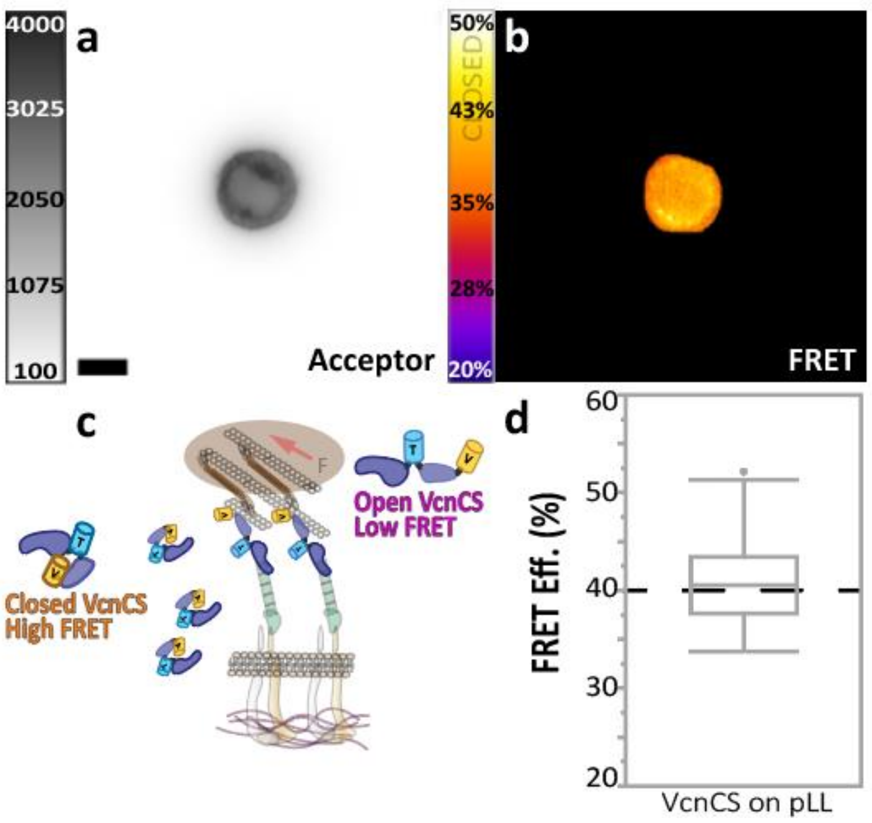

Supplementary Figure 8. WT VcnCS expressing cells plated on poly-L-lysine coated glass show closed VcnCS FRET efficiency.

a, Acceptor intensity (Scale bar: 10 µm) and **b,** FRET efficiency image of Vcn$^{-/-}$ MEF expressing VcnCS, which localizes diffusely in the cytosol when plated on pLL-coated glass **c,** Schematic depicting closed conformation (high FRET) and open conformation (low FRET) of VcnCS in the cytosol **d,** Box-whisker plot of FRET efficiency (n = 62 cells, respectively, from N=3 independent experimental days). Box depicts the median as the center value, the 25th percentile as the lower bound, and the 75th percentile as the upper bound. Whiskers extend 1.5 times the IQR from the bottom and top of box, or to the minimum and maximum of the data if the data does not extend to the whiskers. Source data are provided as a Source Data file.

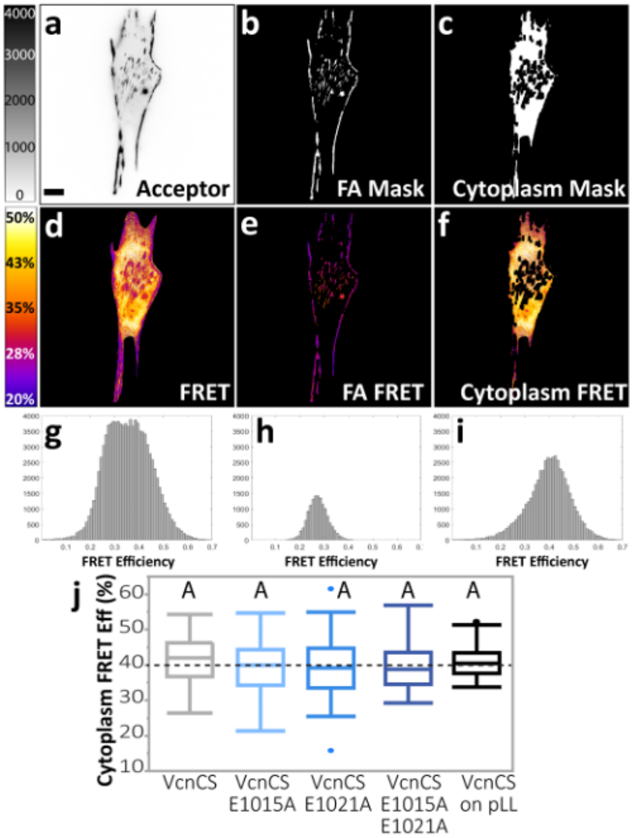

Supplementary Figure 9. WT VcnCS and VcnCS DAFS variants are closed in the cytosol. **a,** Representative acceptor image of Vcn$^{-/-}$ VcnCS expressing MEF. Scale bar: 10 µm. **b,** Segmented FA mask image **c,** Representative cytoplasm mask image generated from dilated FA mask **d,** Representative image of FRET pixels in cell cytoplasm **e,** Representative image of FA FRET pixels, from FA mask applied to FRET **f,** Representative cytoplasmic FRET, from cytoplasm mask applied to cell **g-i,** Histogram of FRET pixels in D-F **j,** Box-whisker plots are shown for FRET efficiency of cytoplasmic VcnCS and DAFS variants of VcnCS, and VcnCS on pLL control (*n* = 47, 50, 53, 63, 62 cells, respectively from N=3 independent experimental days). Box depicts the median as the center value, the 25th percentile as the lower bound, and the 75th percentile as the upper bound. Whiskers extend 1.5 times the IQR from the bottom and top of box, or to the minimum and maximum of the data if the data does not extend to the whiskers. One-way ANOVA paired with a Steel-Dwass post-hoc test was performed for statistical analysis. Different letters denote significant difference at $p<0.05$. See Supplementary Table 12 for a detailed listing of *p*-values. Source data are provided as a Source Data file.

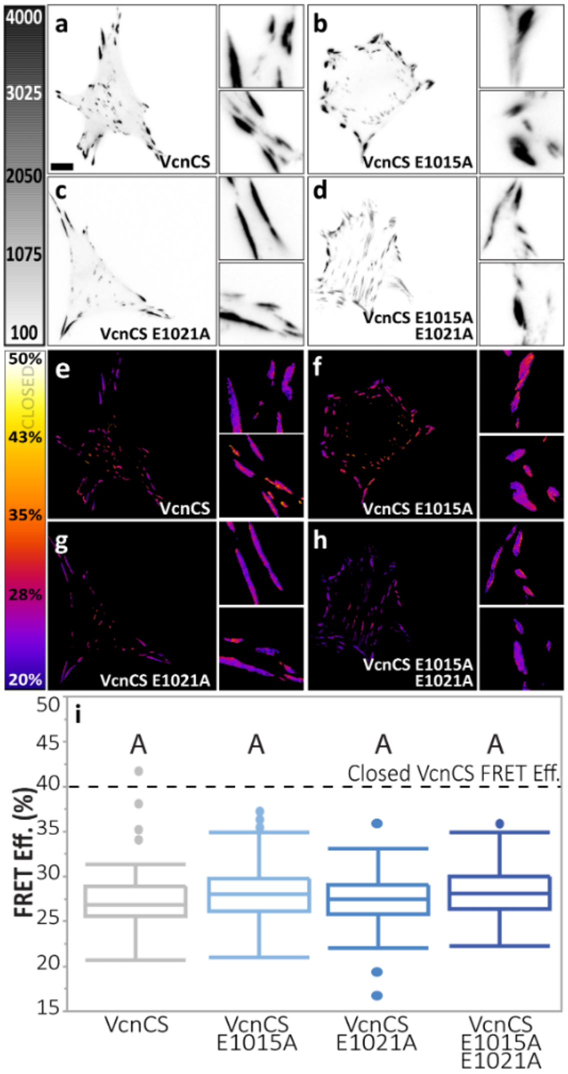

Supplementary Figure 10. WT VcnCS and VcnCS DAFS variants maintain open conformation at FAs.

a-d, FA acceptor intensity (Scale bar: 10 µm) and **e-h,** FA masked FRET efficiency is shown for representative Vcn$^{-/-}$ MEFs expressing WT VcnCS and DAFS variant VcnCS constructs. **i,** Box-whisker plots are shown for cell-averaged FA FRET efficiency of VcnCS and VcnCS DAFS variants (*n* = 76, 94, 99, 109 cells, respectively, from N=5 independent experimental days) compared to closed VcnCS FRET efficiency (dotted line). Box depicts the median as the center value, the 25th percentile as the lower bound, and the 75th percentile as the upper bound. Whiskers extend 1.5 times the IQR from the bottom and top of box, or to the minimum and maximum of the data if the data does not extend to the whiskers. One-way ANOVA paired with Tukey's HSD test was used for statistical analysis. Different letters denote significant difference at $p<0.05$. See Supplementary Table 13 for a detailed listing of *p*-values. Source data are provided as a Source Data file.

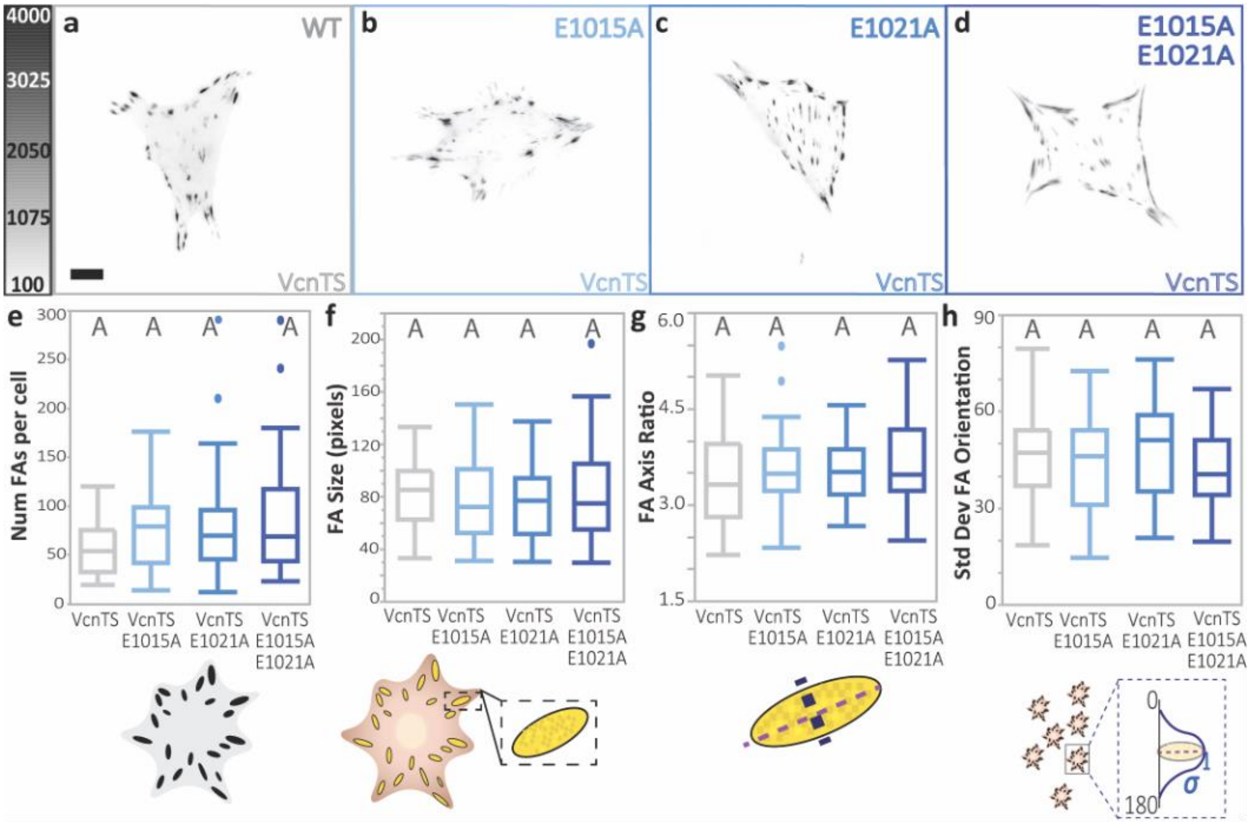

Supplementary Figure 11. VcnTS DAFS Variants do not display altered FA characteristics. **a-d,** Representative images of Vcn$^{-/-}$ MEFs expressing WT VcnTS and VcnTS DAFS variants. WT VcnTS is shown in gray and variants of VcnTS are shown in shades of blue. Scale bar: 10 µm. Quantification of FA morphometric characteristics are shown including **e,** FA number, **f,** FA area, **g,** FA axis ratio, and **h,** standard deviation of FA orientation. Plots shown for WT VcnTS and DAFS variants of VcnTS (*n*= 45, 49, 48, 46 cells, respectively, collected over N=3 independent experiments). Box depicts the median as the center value, the 25th percentile as the lower bound, and the 75th percentile as the upper bound. Whiskers extend 1.5 times the IQR from the bottom and top of box, or to the minimum and maximum of the data if the data does not extend to the whiskers. One-way ANOVA paired with a Steel-Dwass test (e,f,g) or Tukey's HSD test (h)was conducted across all VcnVenus, VcnCS and VcnTS constructs used for statistical analysis. Differing letters denote significant difference at p<0.05. See Supplementary Tables 4-7 for a detailed listing of p-values. Source data are provided as a Source Data file.

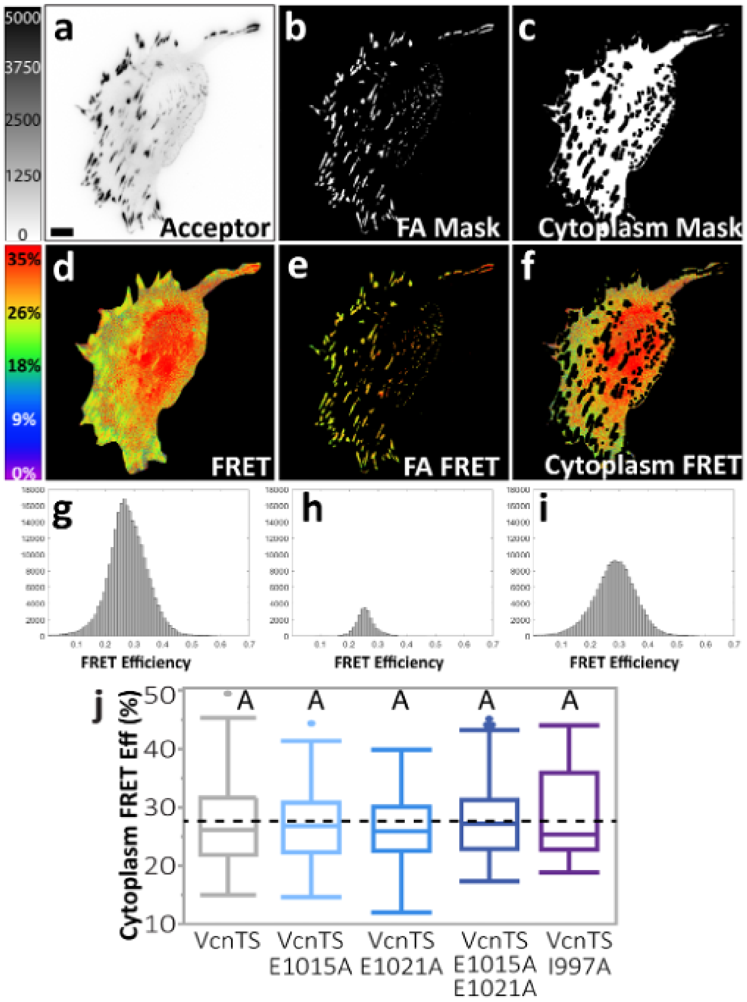

Supplementary Figure 12. VcnTS and VcnTS DAFS variants are unloaded in the cytosol.
a, Representative acceptor image of Vcn$^{-/-}$ VcnTS expressing MEF. Scale bar: 10 µm. **b,** Segmented FA mask image **c,** Representative cytoplasm mask image generated from dilated FA mask **d,** Representative image of FRET pixels in cell cytoplasm and FAs **e,** Representative image of FA FRET pixels, from FA mask applied to FRET **f**, Representative cytoplasmic FRET, from cytoplasm mask applied to cell **g-i,** Histogram of FRET pixels in (d-f) **j,** Box-whisker plots are shown for FRET efficiency of cytoplasmic VcnTS, DAFS residue variants of VcnTS and VcnTS I997A (*n* = 53, 68, 63, 80, 44 cells, respectively from N=4 days). Box depicts the median as the center value, the 25th percentile as the lower bound, and the 75th percentile as the upper bound. Whiskers extend 1.5 times the IQR from the bottom and top of box, or to the minimum and maximum of the data if the data does not extend to the whiskers. One-way ANOVA paired with Tukey post-hoc test was performed for statistical analysis. Different letters denote significant difference at $p<0.05$. See Supplementary Table 14 for a detailed listing of *p*-values. Source data are provided as a Source Data file.

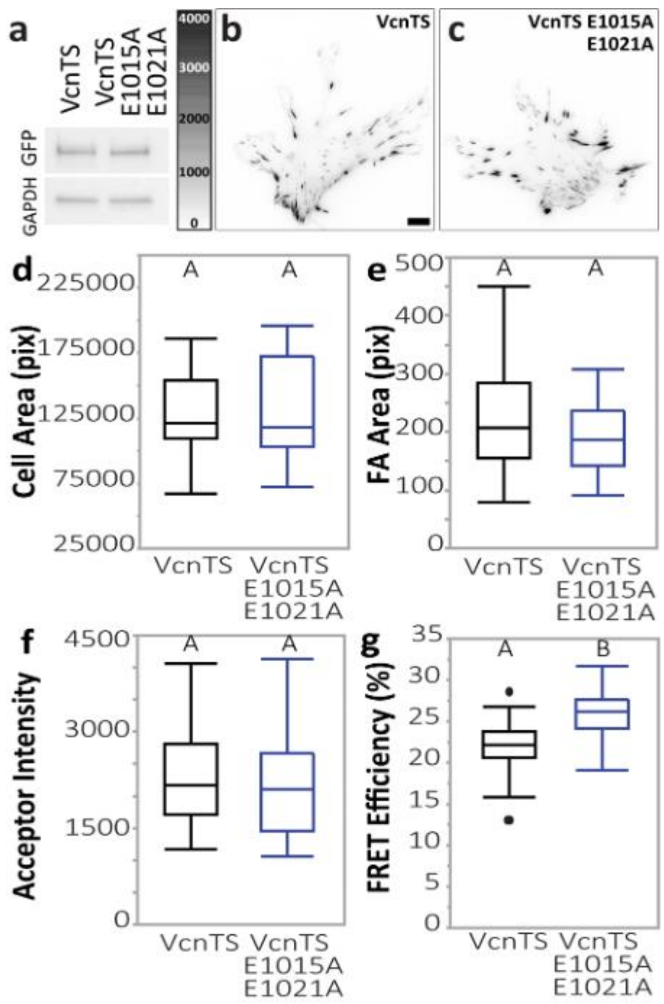

Supplementary Figure 13. Stably expressed VcnTS and VcnTS double DAFS variant characteristics are conserved from transient expression.

a, Western blot of stably expressed VcnTS and VcnTS E1015A-E1021A cell lines, showing presence of variants does not affect VcnTS cellular expression ability. Anti-GFP used to label VcnTS **b-c,** Representative acceptor intensity image for $Vcn^{-/-}$ VcnTS expressing cell and DAFS residue variant $Vcn^{-/-}$ VcnTS E1015A-E1021A expressing cell. Scale bar: 10 μm. Box-whisker plots of **d,** cell area (one-way ANOVA followed by F-test, p=0.4546) **e,** FA area (one-way ANOVA followed by F-test, p=0.1113) **f,** FA Acceptor intensity (one-way ANOVA followed by F-test, p=0.6497) and **g,** FRET efficiency (one-way ANOVA followed by F-test, p=0.0001) for VcnTS population (n=25) and VcnTS E1015A-E1021A population (n=22) selected for FRET-FRAP assay. Differing letters denote significance at $p<0.05$. Box depicts the median as the center value, the 25th percentile as the lower bound, and the 75th percentile as the upper bound. Whiskers extend 1.5 times the IQR from the bottom and top of box, or to the minimum and maximum of the data if the data does not extend to the whiskers. Source data are provided as a Source Data file.

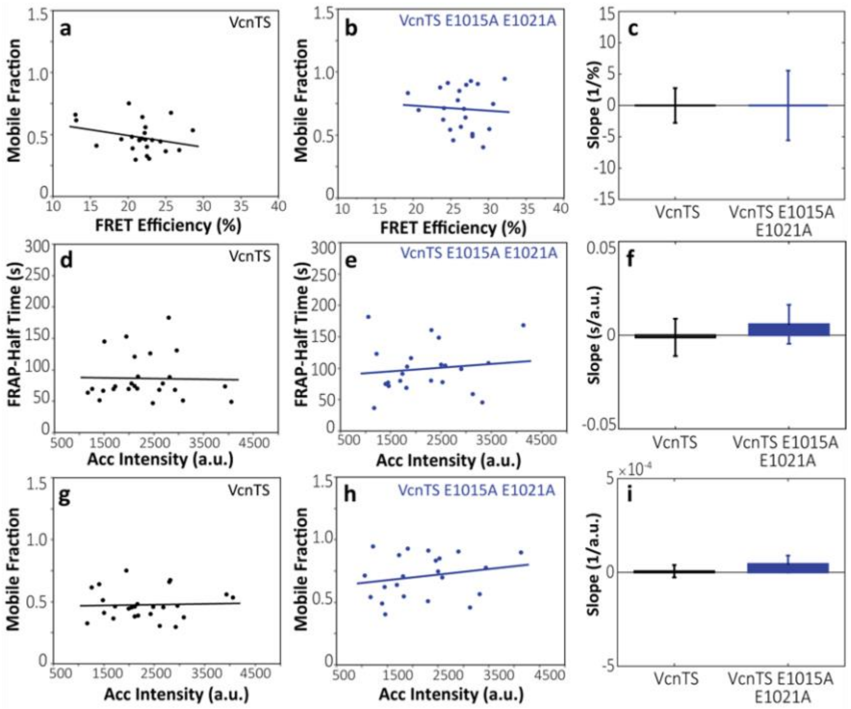

Supplementary Figure 14. Stably expressed VcnTS and VcnTS double DAFS variant dynamics and mobile fraction are independent of acceptor intensity.

a, Correlation between recovery half-time and acceptor intensity for VcnTS (*n*=25, *p*=0.91) and **b,** for VcnTS E1015A-E1021A (*n* = 22, *p*=0.58) **c,** Regression slopes calculated for FRAP half-time vs. Venus mean intensity at the single FA. Bars represent slope and error bars represent standard error of the regression slope. None of the slopes are statistically different from zero, as determined by a one-way ANOVA followed by an F-test **d,** Correlation between mobile fraction and acceptor intensity for VcnTS (*n*=25, *p*=0.85) and **e,** for VcnTS E1015A-E1021A (*n*=22, *p*=0.34) **f,** Regression slopes calculated for mobile fraction vs. Venus mean intensity. A least-squares linear regression was fit to the FRAP mobile fraction and acceptor intensity data for each construct. Bars represent slope and error bars represent standard error of the regression slope. None of the slopes are statistically different from zero, as determined by a one-way ANOVA followed by an F-test. **g,** Correlation between mobile fraction and FRET efficiency for VcnTS (*n*=25, *p*=0.16) and **h,** for VcnTS E1015A-E1021A (*n*=22, *p*=0.75) **i,** Regression slopes calculated for mobile fraction vs. FRET efficiency at the single FA. Bars represent slope and error bars represent standard error of the regression slope. None of the slopes are statistically different from zero, as determined by a one-way ANOVA followed by an F-test. Source data are provided as a Source Data file.

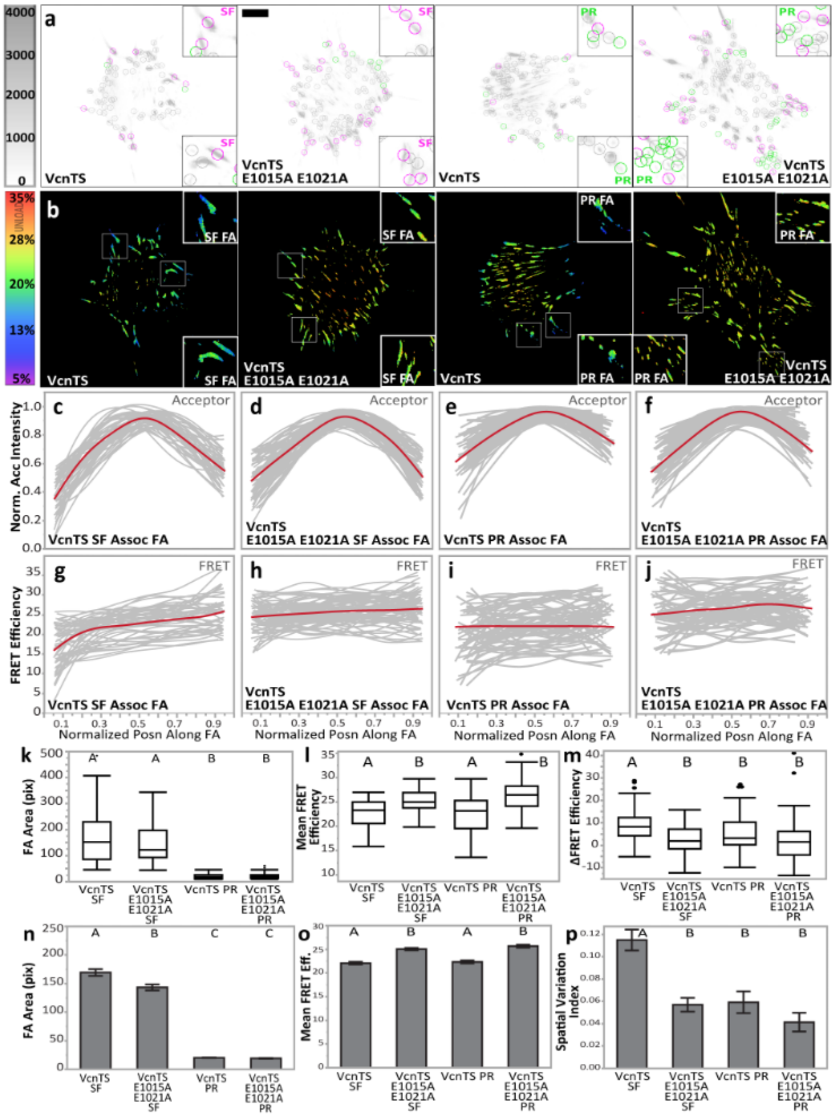

Supplementary Figure 15. VcnTS DAFS double variant SF-associated FAs exhibit loss of characteristic load gradients.

a, Representative images of cells stably expressing VcnTS or VcnTS E1015A-E1021A with machine learning-identified SF-associated or PR-associated FAs. Scale bar: 10 µm. **b,** Corresponding FRET image of cells from panel A, with pictured insets of SF or PR-associated FAs. FA Line scans of normalized acceptor intensity over normalized length of FA for representative **c,** VcnTS SF-associated FAs (*n*=46) **d,** VcnTS E1015A-E1021A SF-associated FAs (*n*=73) **e,** VcnTS PR-associated FAs (*n*=63) **f,** VcnTS E1015A-E1021A PR-associated FAs (*n*=67). FA line scans of FRET efficiency over normalized length of FA for **g,** VcnTS SF-associated FAs (*n*=46) **h,** VcnTS E1015A-E1021A SF-associated(*n*=73) FAs **i,** VcnTS PR-associated FAs (*n*=63) **j,** VcnTS E1015A-E1021A PR-associated FAs (*n*=67) **k,** FA area for representative FAs in (c-j) from N=3 days. Differing letters denote significance p<0.05, one-way ANOVA paired with post-hoc Steel Dwass test. **l,** Mean FA FRET efficiency for FAs in (c-j). Differing letters denote significance at p<0.05, one-way ANOVA paired with post-hoc Steel-

Dwass Test. **M,** Change in FRET efficiency across FA line scan for FAs in (c-j). Differing letters denote significance at $p<0.05$, one-way ANOVA paired with post-hoc Steel-Dwass Test. **n,** Cell averaged FA area in pixels of SF- and PR- associated FAs of all cells expressing VcnTS or VcnTS E1015A-E1021A respectively (VcnTS SF FAs from *n*=102 cells, VcnTS E1015A-E1021A SF FAs from *n*=79 cells, VcnTS PR FAs from *n*= 103 cells, VcnTS E1015A-E1021A PR FAs from *n*= 79 cells). Differing letters denote significance at $p<0.05$, one-way ANOVA paired with Steel-Dwass Test. **o,** Cell averaged FA FRET efficiency of SF- and PR-associated FAs of cells from n, and N= 3 days. Differing letters denote significance at $p<0.05$, one-way ANOVA paired with Steel-Dwass Test**. p,** Cell averaged spatial variation index of SF and PR associated FAs from n, and N=3 days. Differing letters denote significance at $p<0.05$, one-way ANOVA paired with Steel-Dwass Test**.** Bar height represents mean, and error bar represents sample standard error. Box depicts the median as the center value, the 25th percentile as the lower bound, and the 75th percentile as the upper bound. Whiskers extend 1.5 times the IQR from the bottom and top of box, or to the minimum and maximum of the data if the data does not extend to the whiskers. See Supplementary Tables 15-20 for a detailed listing of *p*-values. Source data are provided as a Source Data file.

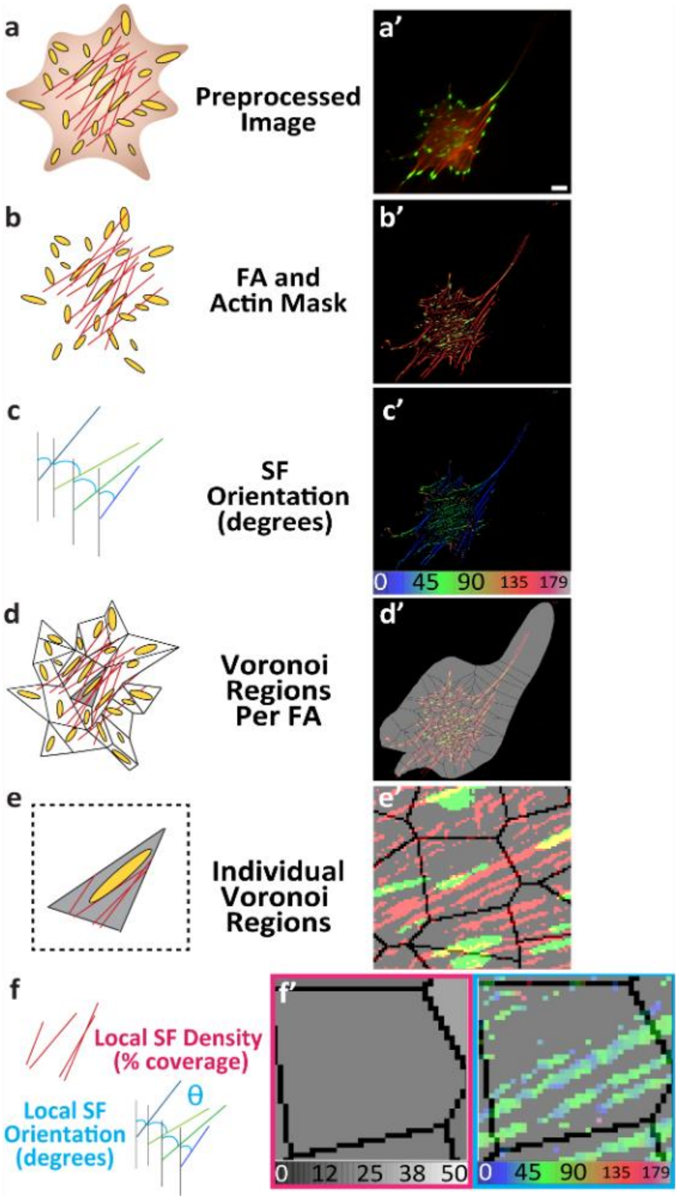

Supplementary Figure 16. Image processing approach for quantifying SF organization metrics.

a, Schematic (scale bar: 10 µm) and **a'** representative background subtracted image of Vcn$^{-/-}$ MEF stably expressing VcnTS, stained for actin (red). **b,** Schematic and **b'** representative image of merged actin mask (red) and FA mask (green). **c,** Schematic and **c'** representative image of SF orientations within the whole cell **d,** Schematic and **d'** representative image of Voronoi regions overlay atop B'. **e,** Schematic and **e'** representative image of zoomed in, singular Voronoi region overlay atop B'. **f,** Schematics and **f'** representative map of local SF density percentage and SF orientation per Voronoi region.

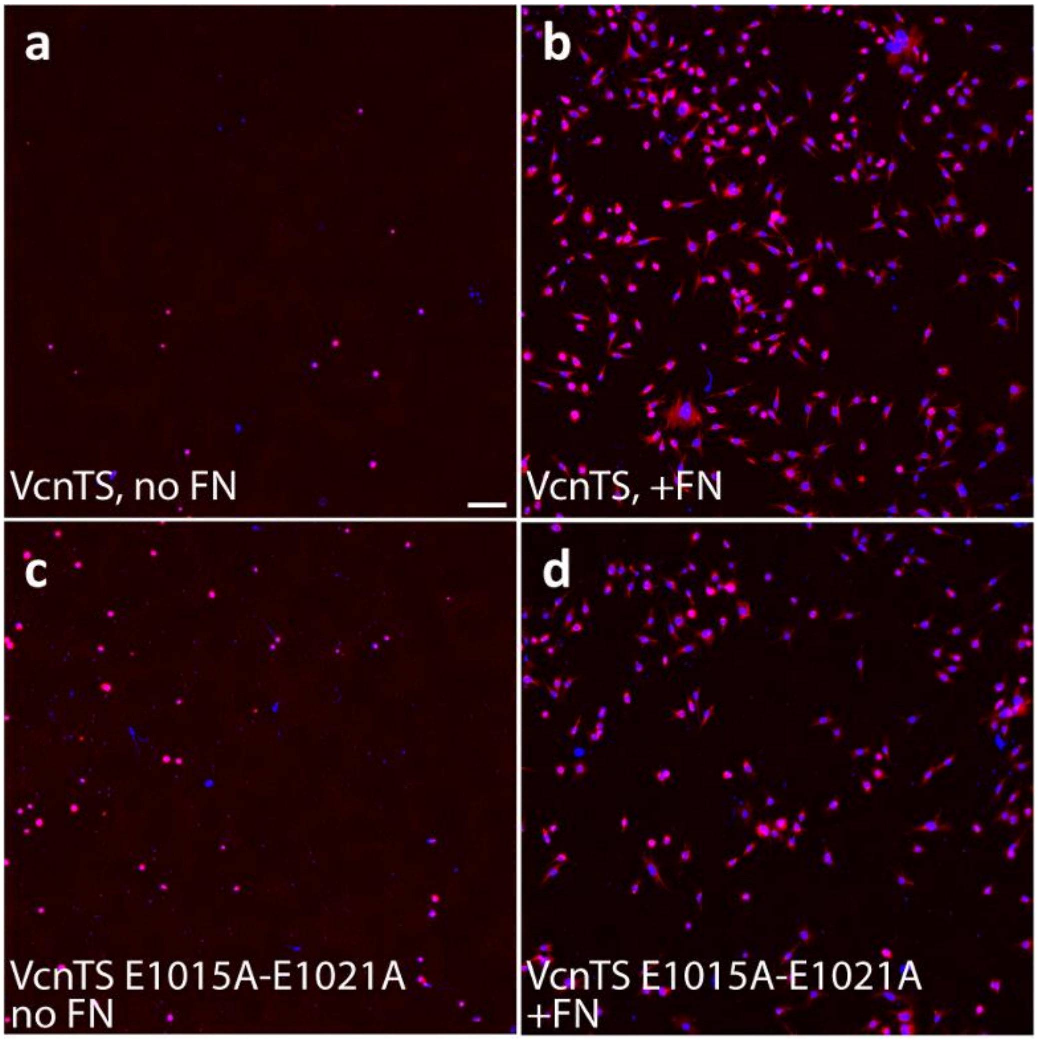

Supplementary Figure 17 (Figure 8 Additional Material): Western and representative Boyden chamber assay images.

Representative fields of view from haptotactic cell migration assay shown in **figure 8g**. Scale bar: 100 μm. **a,** $Vcn^{-/-}$MEF VcnTS MEFs migrating with no FN gradient, **b,** $Vcn^{-/-}$MEF VcnTS MEFs migrating with FN gradient **c,** $Vcn^{-/-}$MEF VcnTS E1015A-E1021A MEFs migrating with no FN gradient **d,** $Vcn^{-/-}$MEF VcnTS E1015A-E1021A MEFs migrating with FN gradient.

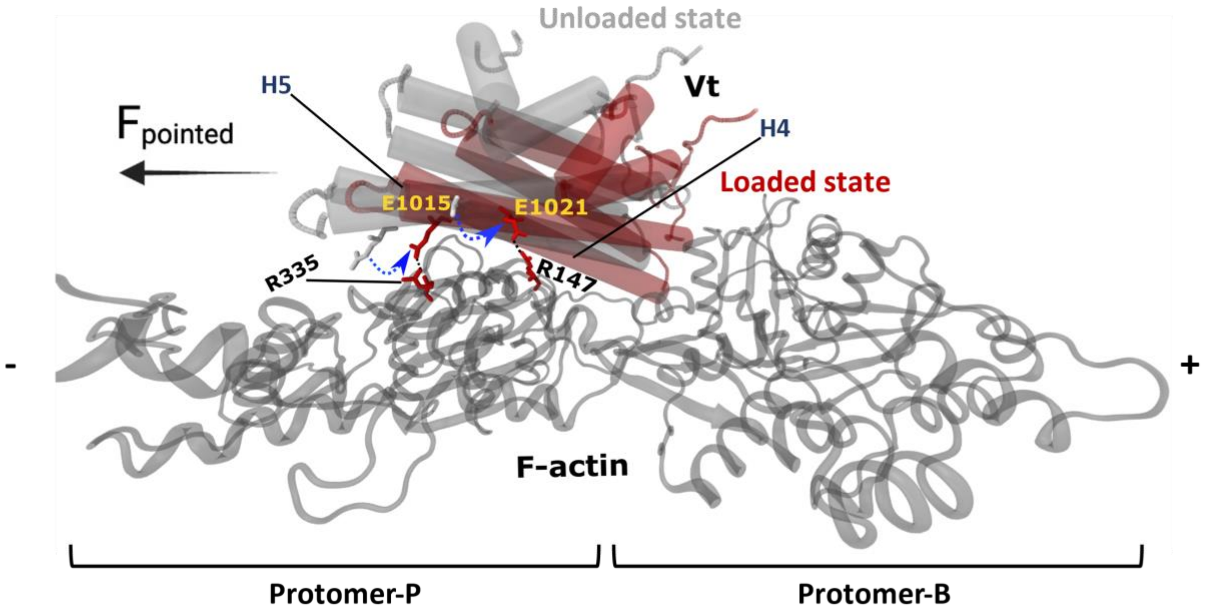

Supplementary Figure 18. $F_{pointed}$-directed pulling force induces structural changes in the Vt helical bundle to strengthen existing and new catch bonds between F-actin and Vt.
DMD pulling simulation trajectories depict twisting and conformational reorientation of helix-4 and helix-5 of Vt in the $F_{pointed}$ direction that exposes side chains of E1015 and E1021 to generate a new actin binding interface. These structural changes also reinforce existing H-bonds between helix-4 of Vt and F-actin. Color scheme: Unloaded Vt in light gray, force-loaded Vt in red, and F-actin in dark gray. The catch bond forming residue pairs are shown in licorice representation.

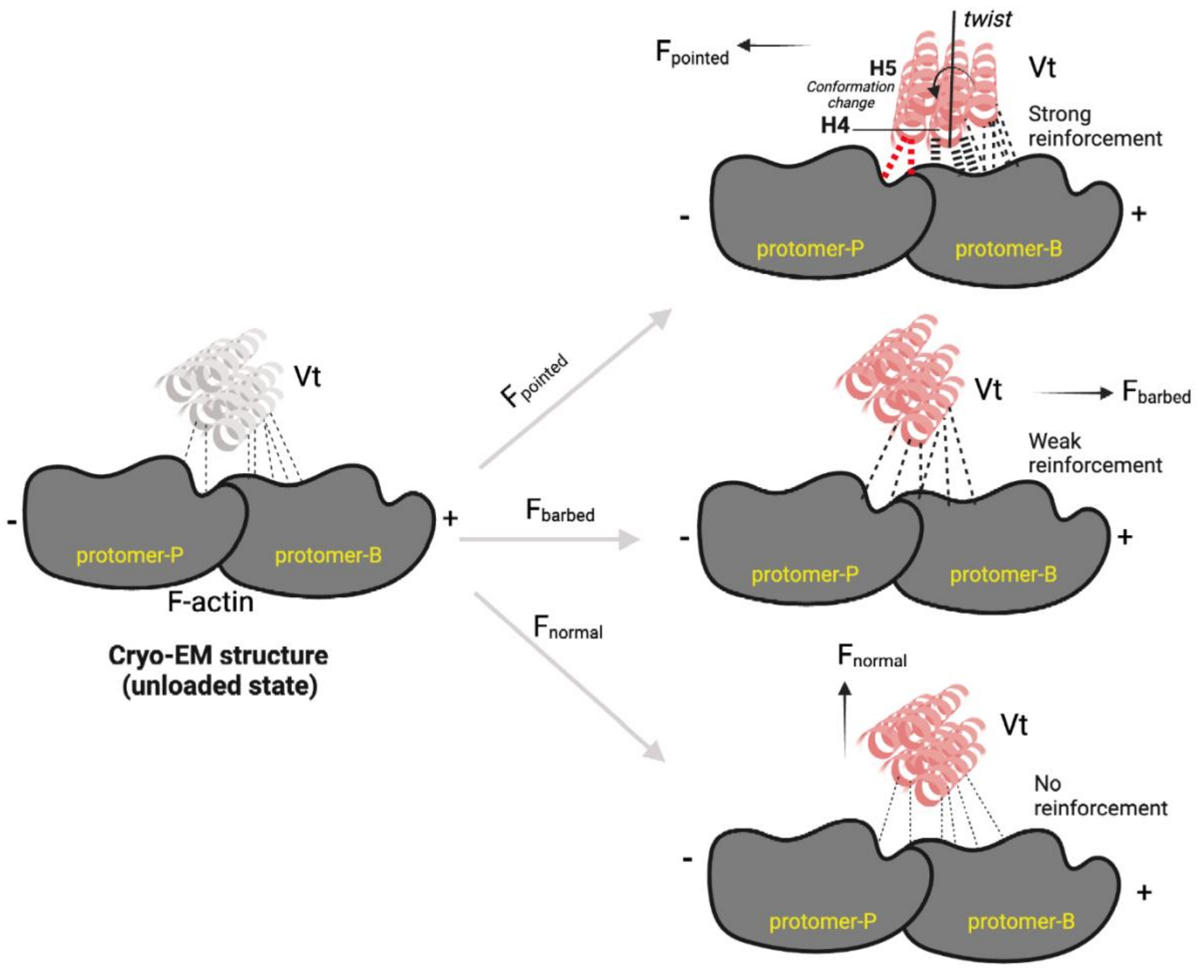

Supplementary Figure 19. A schematic representation of directional catch bonding in Vcn. Force-induced structural changes in Vt facilitate formation of directionally asymmetric catch bonds with F-actin. Specifically, $F_{pointed}$ directed pulling force in the range 124 – 128 pN induces a twisting rearrangement of the Vt helical bundle along the short-axis of the actin filament. This twisting motion generates a new binding interface at the C-terminal end of helix-4 and helix-5 that promotes formation of new H-bond interactions as well as strengthens existing H-bonds. In contrast, the strength of existing H-bonds in F_{barbed} trajectories was reduced relative to $F_{pointed}$, whereas only weak and transient interactions were observed in F_{normal} trajectories.

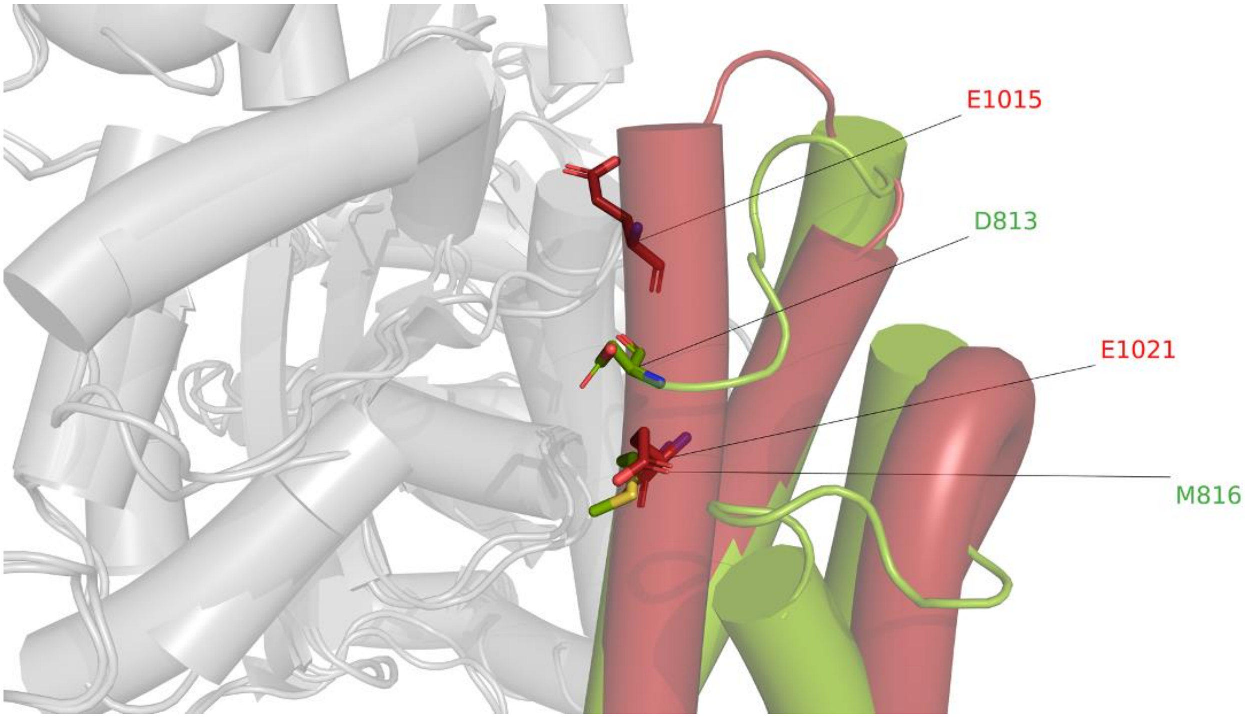

Supplementary Figure 20. Structural alignment of Catn:F-actin and Vt:F-actin complexes. Structural superimposition of Catn:F-actin (PDB ID: 6UPV) and Vt:F-actin complex (PDB ID: 3JBI) cryo-EM reconstructions highlighting key residues involved in intermolecular interactions. Catn is shown in green, Vt in red, and F-actin in gray. Key residues involved in intermolecular interactions, Catn: D813, M816 and Vt: E1015, E1021, are shown as stick models.

Supplementary Table 1:

Actin Binding Statistical Significance (one-way ANOVA, post-hoc Tukey test)		
**Level**	**- Level**	**p-Value**
Vt	No Actin	<.0001
Vt E1015AE1021A	No Actin	<.0001
Vt E1015A	No Actin	<.0001
Vt E1021A	No Actin	<.0001
I997A	No Actin	<.0001
Vt	I997A	<.0001
Vt E1015AE1021A	I997A	<.0001
Vt E1015A	I997A	<.0001
Vt E1021A	I997A	<.0001
Vt	Vt E1021A	0.003
Vt	Vt E1015A	0.026
Vt	Vt E1015AE1021A	0.2384
Vt E1015AE1021A	Vt E1021A	0.5578
Vt E1015AE1021A	Vt E1015A	0.931
Vt E1015A	Vt E1021A	0.9795

Supplementary Table 2:

Actin Bundling Statistical Significance (one-way ANOVA, post-hoc Tukey test)		
**Level**	**- Level**	**p-Value**
Vt E1021A	Actin Only	<.0001
Vt	Actin Only	<.0001
Vt E1015A E1021A	Actin Only	<.0001
Vt E1015A	Actin Only	<.0001
Vt E1021A	Vt I997A	<.0001
Vt	Vt I997A	<.0001
Vt E1015A E1021A	Vt I997A	<.0001
Vt E1015A	Vt I997A	<.0001
Vt I997A	Actin Only	<.0001
Vt E1021A	Vt E1015A	0.9894
Vt E1021A	Vt E1015A E1021A	0.9987
Vt	Vt E1015A	0.9988
Vt E1021A	Vt	0.9999
Vt E1015A E1021A	Vt E1015A	0.9999
Vt	Vt E1015A E1021A	1

Supplementary Table 3:

PIP2 Binding Statistical Significance (one-way ANOVA, post-hoc Tukey test)		
**Level**	**- Level**	**p-Value**
E1015A-E1021A	Vt	0.2465
E1015A	Vt	0.4836
E1015A-E1021A	E1021A	0.6671
E1021A	Vt	0.8641
E1015A	E1021A	0.9072
E1015A-E1021A	E1015A	0.9643

Supplementary Table 4:

Number FAs per Cell Statistical Significance (one-way ANOVA, post-hoc Steel-Dwass test)		
**Level**	**- Level**	***p*-value**
VcnVenus E1015A	VcnTS	0.2509
VcnTS E1015A E1021A	VcnTS	0.2771
VcnVenus E1015A	VcnVenus	0.3585
VcnVenus E1015A	VcnCS E1021A	0.4202
VcnTS E1015A E1021A	VcnCS E1021A	0.5341
VcnTS E1015A	VcnTS	0.7828
VcnTS E1021A	VcnTS	0.8601
VcnVenus E1015A E1021A	VcnTS	0.9145
VcnTS E1015A	VcnCS E1021A	0.9204
VcnTS E1021A	VcnCS E1021A	0.9315
VcnVenus E1015A E1021A	VcnCS E1021A	0.9572
VcnVenus E1015A E1021A	VcnVenus	0.9733
VcnVenus E1015A	VcnCS E1015A	0.9979
VcnVenus E1015A	VcnTS E1021A	0.9985
VcnVenus E1015A	VcnTS E1015A	0.9998
VcnTS E1015A E1021A	VcnCS E1015A	0.9996
VcnTS E1015A E1021A	VcnCS	0.9995
VcnVenus E1015A	VcnCS	0.9997
VcnTS E1015A E1021A	VcnTS E1015A	0.9998
VcnVenus E1015A	VcnCS E1015A E1021A	1
VcnTS E1015A E1021A	VcnCS E1015A E1021A	1
VcnCS E1015A E1021A	VcnCS	1
VcnCS E1015A E1021A	VcnCS E1015A	1
VcnVenus	VcnTS	1
VcnTS E1015A	VcnCS	1
VcnTS E1015A	VcnCS E1015A	1
VcnVenus E1015A	VcnTS E1015A E1021A	1
VcnVenus	VcnCS E1021A	1
VcnCS E1015A	VcnCS	1
VcnVenus E1021A	VcnTS	1
VcnTS E1021A	VcnCS E1015A	1
VcnVenus E1021A	VcnCS E1021A	1
VcnTS	VcnCS E1021A	1
VcnTS E1021A	VcnCS	1
VcnVenus E1021A	VcnVenus	1
VcnVenus E1015A E1021A	VcnTS E1021A	1

VcnTS E1021A	VcnTS E1015A	1
VcnTS E1015A	VcnCS E1015A E1021A	1
VcnVenus E1015A E1021A	VcnCS	1
VcnVenus E1015A E1021A	VcnCS E1015A	1
VcnTS E1021A	VcnCS E1015A E1021A	1
VcnVenus E1015A E1021A	VcnTS E1015A	1
VcnVenus E1015A E1021A	VcnCS E1015A E1021A	0.9998
VcnTS E1021A	VcnTS E1015A E1021A	0.9993
VcnVenus E1015A E1021A	VcnTS E1015A E1021A	0.9891
VcnVenus E1015A E1021A	VcnVenus E1015A	0.9866
VcnCS E1021A	VcnCS	0.9707
VcnCS E1021A	VcnCS E1015A	0.9688
VcnVenus	VcnCS	0.9504
VcnVenus	VcnCS E1015A	0.9432
VcnVenus	VcnTS E1021A	0.9325
VcnTS	VcnCS E1015A	0.8861
VcnTS	VcnCS	0.8683
VcnVenus E1021A	VcnCS	0.9273
VcnVenus E1021A	VcnCS E1015A	0.9077
VcnVenus	VcnTS E1015A	0.8602
VcnVenus E1021A	VcnVenus E1015A E1021A	0.849
VcnVenus E1021A	VcnTS E1021A	0.793
VcnVenus E1021A	VcnTS E1015A	0.7855
VcnCS E1021A	VcnCS E1015A E1021A	0.7356
VcnVenus	VcnCS E1015A E1021A	0.7155
VcnTS	VcnCS E1015A E1021A	0.5812
VcnVenus	VcnTS E1015A E1021A	0.4777
VcnVenus E1021A	VcnCS E1015A E1021A	0.4582
VcnVenus E1021A	VcnTS E1015A E1021A	0.3297
VcnVenus E1021A	VcnVenus E1015A	0.192

Supplementary Table 5:

FA Size Statistical Significance (one-way ANOVA, post-hoc Steel-Dwass test)		
**Level**	**- Level**	***p*-value**
VcnCS E1015A E1021A	VcnCS E1015A	0.9758
VcnCS E1021A	VcnCS E1015A	0.9952
VcnTS	VcnCS E1015A	0.9996
VcnTS E1015A E1021A	VcnTS E1015A	0.9999
VcnVenus E1015A	VcnVenus	1
VcnTS E1015A E1021A	VcnCS E1015A	1
VcnVenus E1021A	VcnVenus E1015A E1021A	1
VcnCS E1015A E1021A	VcnCS	1
VcnCS E1021A	VcnCS	1
VcnTS	VcnCS	1
VcnVenus E1015A	VcnTS E1021A	1
VcnVenus E1015A	VcnCS E1015A	1
VcnVenus E1015A	VcnTS E1015A	1
VcnTS	VcnCS E1021A	1
VcnTS E1015A	VcnCS E1015A	1
VcnTS E1015A E1021A	VcnTS	1
VcnTS E1015A E1021A	VcnCS	1
VcnCS E1021A	VcnCS E1015A E1021A	1
VcnTS E1021A	VcnTS E1015A	1
VcnTS E1021A	VcnCS E1015A	1
VcnTS	VcnCS E1015A E1021A	1
VcnTS E1015A E1021A	VcnCS E1021A	1
VcnVenus E1021A	VcnVenus	1
VcnVenus	VcnTS E1015A	1
VcnVenus	VcnCS E1015A	1
VcnTS E1015A E1021A	VcnCS E1015A E1021A	1
VcnCS E1015A	VcnCS	0.9999
VcnVenus	VcnTS E1021A	0.9999
VcnVenus E1015A E1021A	VcnVenus	0.9998
VcnVenus E1021A	VcnCS E1015A	0.9997
VcnTS E1015A	VcnCS	0.9994
VcnVenus E1021A	VcnTS E1015A	0.9995
VcnVenus E1015A	VcnTS E1015A E1021A	0.999
VcnVenus E1015A	VcnCS	0.9975
VcnTS E1015A	VcnTS	0.9978
VcnTS E1021A	VcnTS E1015A E1021A	0.997

VcnVenus E1015A E1021A	VcnCS E1015A	0.9944
VcnVenus E1015A E1021A	VcnTS E1015A	0.9926
VcnTS E1021A	VcnTS	0.9876
VcnVenus E1015A E1021A	VcnTS E1021A	0.99
VcnTS E1021A	VcnCS	0.9772
VcnTS E1015A	VcnCS E1021A	0.9838
VcnVenus	VcnTS E1015A E1021A	0.9796
VcnVenus E1021A	VcnVenus E1015A	0.9893
VcnVenus E1021A	VcnTS E1021A	0.9877
VcnVenus E1015A E1021A	VcnVenus E1015A	0.9745
VcnVenus E1015A	VcnTS	0.9652
VcnTS E1021A	VcnCS E1021A	0.9618
VcnTS E1015A	VcnCS E1015A E1021A	0.9698
VcnVenus E1015A	VcnCS E1021A	0.9557
VcnTS E1021A	VcnCS E1015A E1021A	0.953
VcnVenus	VcnCS	0.8868
VcnVenus E1021A	VcnTS E1015A E1021A	0.8364
VcnVenus	VcnCS E1021A	0.7409
VcnVenus E1015A	VcnCS E1015A E1021A	0.8018
VcnVenus E1021A	VcnCS	0.7661
VcnVenus	VcnTS	0.6816
VcnVenus E1015A E1021A	VcnTS E1015A E1021A	0.6903
VcnVenus E1015A E1021A	VcnCS	0.6255
VcnVenus	VcnCS E1015A E1021A	0.6865
VcnVenus E1015A E1021A	VcnCS E1021A	0.2393
VcnVenus E1015A E1021A	VcnTS	0.2108
VcnVenus E1021A	VcnCS E1021A	0.2407
VcnVenus E1021A	VcnTS	0.1847
VcnVenus E1015A E1021A	VcnCS E1015A E1021A	0.1189
VcnVenus E1021A	VcnCS E1015A E1021A	0.0782

Supplementary Table 6:

FA Axis Ratio Statistical Significance (one-way ANOVA, post-hoc Steel-Dwass test)		
**Level**	**- Level**	***p*-value**
VcnVenus E1015A	VcnCS E1015A	0.1181
VcnTS E1015A E1021A	VcnCS E1015A	0.0906
VcnTS E1021A	VcnCS E1015A	0.1821
VcnVenus E1021A	VcnCS E1015A	0.2874
VcnTS E1015A E1021A	VcnCS E1021A	0.2217
VcnTS E1015A	VcnCS E1015A	0.2654
VcnVenus E1015A	VcnCS E1021A	0.4011
VcnVenus	VcnCS E1015A	0.3296
VcnTS E1021A	VcnCS E1021A	0.4418
VcnTS E1015A	VcnCS E1021A	0.5936
VcnVenus E1021A	VcnCS E1021A	0.7173
VcnVenus	VcnCS E1021A	0.6107
VcnTS E1015A E1021A	VcnCS E1015A E1021A	0.7566
VcnTS E1015A E1021A	VcnCS	0.6473
VcnVenus E1015A	VcnCS	0.753
VcnVenus E1021A	VcnVenus E1015A E1021A	0.9257
VcnCS E1015A E1021A	VcnCS E1015A	0.9119
VcnVenus E1015A	VcnCS E1015A E1021A	0.9485
VcnTS	VcnCS E1015A	0.9097
VcnTS E1021A	VcnCS	0.9275
VcnTS E1015A E1021A	VcnTS	0.9605
VcnVenus E1015A E1021A	VcnCS E1015A	0.9722
VcnTS E1015A	VcnCS	0.9678
VcnVenus E1021A	VcnCS	0.9833
VcnVenus E1021A	VcnCS E1015A E1021A	0.9939
VcnVenus	VcnCS	0.9896
VcnTS E1021A	VcnCS E1015A E1021A	0.9958
VcnTS	VcnCS E1021A	0.9944
VcnTS E1015A	VcnCS E1015A E1021A	0.9979
VcnVenus E1015A	VcnTS	0.9975
VcnTS E1015A E1021A	VcnTS E1015A	0.9997
VcnVenus E1015A E1021A	VcnCS E1021A	0.9998
VcnTS E1021A	VcnTS	0.9999
VcnVenus	VcnCS E1015A E1021A	0.9999
VcnVenus E1015A	VcnVenus	0.9999
VcnVenus E1021A	VcnTS	1

VcnCS E1021A	VcnCS E1015A	1
VcnTS E1015A	VcnTS	1
VcnVenus	VcnTS	1
VcnVenus E1015A	VcnTS E1015A	1
VcnTS	VcnCS	1
VcnVenus E1021A	VcnVenus	1
VcnVenus E1015A	VcnTS E1021A	1
VcnCS E1015A E1021A	VcnCS	1
VcnTS	VcnCS E1015A E1021A	1
VcnTS E1021A	VcnTS E1015A	1
VcnVenus E1021A	VcnTS E1015A	1
VcnVenus E1015A E1021A	VcnCS	1
VcnVenus E1021A	VcnTS E1021A	1
VcnVenus	VcnTS E1015A	1
VcnVenus E1015A E1021A	VcnTS	1
VcnVenus E1015A E1021A	VcnCS E1015A E1021A	1
VcnCS E1021A	VcnCS	1
VcnVenus	VcnTS E1021A	1
VcnVenus E1015A	VcnTS E1015A E1021A	1
VcnVenus E1021A	VcnVenus E1015A	1
VcnTS E1021A	VcnTS E1015A E1021A	0.9999
VcnCS E1015A	VcnCS	0.997
VcnVenus	VcnTS E1015A E1021A	0.9978
VcnCS E1021A	VcnCS E1015A E1021A	0.9978
VcnVenus E1021A	VcnTS E1015A E1021A	0.998
VcnVenus E1015A E1021A	VcnVenus	0.9491
VcnVenus E1015A E1021A	VcnTS E1015A	0.9402
VcnVenus E1015A E1021A	VcnTS E1021A	0.8633
VcnVenus E1015A E1021A	VcnVenus E1015A	0.7148
VcnVenus E1015A E1021A	VcnTS E1015A E1021A	0.6394

Supplementary Table 7:

Std Dev FA Orientation Statistical Significance (one-way ANOVA, post-hoc Tukey test)		
**Level**	**- Level**	***p*-value**
VcnTS E1021A	VcnTS E1015A E1021A	0.3281
VcnTS E1021A	VcnTS E1015A	0.5701
VcnTS E1021A	VcnVenus E1015A E1021A	0.8115
VcnTS	VcnTS E1015A E1021A	0.8902
VcnTS E1021A	VcnVenus E1015A	0.9277
VcnCS	VcnTS E1015A E1021A	0.9723
VcnTS E1021A	VcnCS E1021A	0.9615
VcnTS E1021A	VcnVenus E1021A	0.9586
VcnCS E1015A E1021A	VcnTS E1015A E1021A	0.9708
VcnTS	VcnTS E1015A	0.9801
VcnTS E1021A	VcnVenus	0.9811
VcnCS E1015A	VcnTS E1015A E1021A	0.9876
VcnTS E1021A	VcnCS E1015A	0.9874
VcnVenus	VcnTS E1015A E1021A	0.9879
VcnTS E1021A	VcnCS E1015A E1021A	0.9856
VcnVenus E1021A	VcnTS E1015A E1021A	0.9856
VcnCS E1021A	VcnTS E1015A E1021A	0.9946
VcnTS E1021A	VcnCS	0.9973
VcnCS	VcnTS E1015A	0.9976
VcnVenus E1015A	VcnTS E1015A E1021A	0.9966
VcnCS E1015A E1021A	VcnTS E1015A	0.998
VcnTS	VcnVenus E1015A E1021A	0.9988
VcnCS E1015A	VcnTS E1015A	0.9994
VcnVenus	VcnTS E1015A	0.9995
VcnTS E1021A	VcnTS	0.9995
VcnVenus E1021A	VcnTS E1015A	0.9995
VcnVenus E1015A E1021A	VcnTS E1015A E1021A	0.9998
VcnCS E1021A	VcnTS E1015A	0.9999
VcnCS	VcnVenus E1015A E1021A	1
VcnTS	VcnVenus E1015A	1
VcnVenus E1015A	VcnTS E1015A	1
VcnCS E1015A E1021A	VcnVenus E1015A E1021A	1
VcnTS	VcnCS E1021A	1
VcnCS E1015A	VcnVenus E1015A E1021A	1
VcnTS	VcnVenus E1021A	1
VcnVenus	VcnVenus E1015A E1021A	1

VcnTS	VcnVenus	1
VcnVenus E1021A	VcnVenus E1015A E1021A	1
VcnCS	VcnVenus E1015A	1
VcnTS	VcnCS E1015A	1
VcnTS E1015A	VcnTS E1015A E1021A	1
VcnVenus E1015A E1021A	VcnTS E1015A	1
VcnCS E1021A	VcnVenus E1015A E1021A	1
VcnTS	VcnCS E1015A E1021A	1
VcnCS	VcnCS E1021A	1
VcnCS E1015A E1021A	VcnVenus E1015A	1
VcnVenus E1015A	VcnVenus E1015A E1021A	1
VcnCS	VcnVenus E1021A	1
VcnCS E1015A	VcnVenus E1015A	1
VcnTS	VcnCS	1
VcnCS E1015A E1021A	VcnCS E1021A	1
VcnCS	VcnVenus	1
VcnVenus	VcnVenus E1015A	1
VcnCS	VcnCS E1015A	1
VcnVenus E1021A	VcnVenus E1015A	1
VcnCS E1015A	VcnCS E1021A	1
VcnCS E1015A E1021A	VcnVenus E1021A	1
VcnVenus	VcnCS E1021A	1
VcnCS	VcnCS E1015A E1021A	1
VcnCS E1015A E1021A	VcnVenus	1
VcnCS E1021A	VcnVenus E1015A	1
VcnCS E1015A	VcnVenus E1021A	1
VcnVenus E1021A	VcnCS E1021A	1
VcnCS E1015A E1021A	VcnCS E1015A	1
VcnVenus	VcnVenus E1021A	1
VcnCS E1015A	VcnVenus	1

Supplementary Table 8:

VcnTS FA FRET Statistical Significance (one-way ANOVA, post-hoc Steel-Dwass test)		
**Level**	**- Level**	***p*-value**
TSMod	VcnTS	<.0001
VcnTS I997A	VcnTS	<.0001
VcnTS E1015A E1021A	VcnTS	<.0001
TSMod	VcnTS E1015A	<.0001
TSMod	VcnTS E1021A	<.0001
VcnTS I997A	VcnTS E1015A	<.0001
VcnTS I997A	VcnTS E1021A	<.0001
VcnTS E1021A	VcnTS	0.0011
VcnTS E1015A E1021A	VcnTS E1015A	<.0001
VcnTS I997A	VcnTS E1015A E1021A	<.0001
VcnTS E1015A E1021A	VcnTS E1021A	0.0016
TSMod	VcnTS E1015A E1021A	0.003
VcnTS E1015A	VcnTS	0.5802
VcnTS E1021A	VcnTS E1015A	0.3845
TSMod	VcnTS I997A	0.2354

Supplementary Table 9:

Migrated Cell Count Statistical Significance (one-way ANOVA, post-hoc Steel-Dwass test)		
**Level**	**- Level**	***p*-value**
VcnTS	VcnTS_noFN	<.0001
VcnTS	VcnTS E1015A E1021A_noFN	<.0001
VcnTS	VcnTS E1015A E1021A	<.0001
VcnTS E1015A E1021A	VcnTS_noFN	<.0001
VcnTS E1015A E1021A	VcnTS E1015A E1021A_noFN	<.0001
VcnTS E1015A E1021A_noFN	VcnTS_noFN	0.9997

Supplementary Table 10:

PC/PE Binding Statistical Significance (one-way ANOVA, post-hoc Tukey test)		
**Level**	**- Level**	**p-Value**
E1015A-E1021A	E1021A	0.0027
E1015A-E1021A	Vt	0.0035
E1015A-E1021A	E1015A	0.0152
E1015A	E1021A	0.886
E1015A	Vt	0.9259
Vt	E1021A	0.9995

Supplementary Table 11:

PS Lipids Binding Statistical Significance (one-way ANOVA, post-hoc Tukey test)		
**Level**	**- Level**	**p-Value**
Vt	E1015A	0.0579
Vt	E1015A-E1021A	0.1852
Vt	E1021A	0.4523
E1021A	E1015A	0.64
E1015A-E1021A	E1015A	0.931
E1021A	E1015A-E1021A	0.9347

Supplementary Table 12:

VcnCS Cytosol FRET Statistical Significance (one-way ANOVA, post-hoc Steel-Dwass test)		
**Level**	**- Level**	***p*-value**
VcnCS on pLL	VcnCS E1015A E1021A	0.4679
VcnCS on pLL	VcnCS E1021A	0.6001
VcnCS on pLL	VcnCS E1015A	0.8154
VcnCS on pLL	VcnCS	1
VcnCS E1015A E1021A	VcnCS E1021A	0.9999
VcnCS E1021A	VcnCS E1015A	0.9976
VcnCS E1015A E1021A	VcnCS E1015A	0.9899
VcnCS E1015A	VcnCS	0.8605
VcnCS E1021A	VcnCS	0.6773
VcnCS E1015A E1021A	VcnCS	0.5608

Supplementary Table 13:

VcnCS FA FRET Statistical Significance (one-way ANOVA, post-hoc Tukey test)		
**Level**	**- Level**	***p*-value**
VcnCS E1015A	VcnCS	0.2689
VcnCS E1015A E1021A	VcnCS	0.2532
VcnCS E1015A	VcnCS E1021A	0.3247
VcnCS E1015A E1021A	VcnCS E1021A	0.3056
VcnCS E1021A	VcnCS	0.9958
VcnCS E1015A	VcnCS E1015A E1021A	1

Supplementary Table 14:

VcnTS Cytosol FRET Statistical Significance (one-way ANOVA, post-hoc Tukey test)		
**Level**	**- Level**	***p*-value**
VcnTS I997A	VcnTS E1021A	0.7265
VcnTS I997A	VcnTS E1015A	0.8934
VcnTS E1015A E1201A	VcnTS E1021A	0.8845
VcnTS	VcnTS E1021A	0.9514
VcnTS I997A	VcnTS	0.9834
VcnTS E1015A E1201A	VcnTS E1015A	0.9834
VcnTS I997A	VcnTS E1015A E1201A	0.9904
VcnTS E1015A	VcnTS E1021A	0.9951
VcnTS	VcnTS E1015A	0.9963
VcnTS E1015A E1201A	VcnTS	0.9999

Supplementary Table 15:

**FA Area (of Line Scan Representative FAs) Statistical Significance (one-way ANOVA, post-hoc Tukey Test)**		
**Level**	**- Level**	***p*-value**
VcnTS PR	VcnTS SF	0.9326
VcnTS PR	VcnTS E1015A E1021A PR	<.0001
VcnTS E1015A E1021A SF	VcnTS SF	0.5662
VcnTS E1015A E1021A SF	VcnTS E1015A E1021A PR	<.0001
VcnTS PR	VcnTS E1015A E1021A SF	<.0001
VcnTS E1015A E1021A PR	VcnTS SF	<.0001

Supplementary Table 16:

**Mean FRET Eff (of Line Scan Representative FAs) Statistical Significance (one-way ANOVA, post-hoc Steel Dwass test)**		
**Level**	**- Level**	***p*-value**
VcnTS E1015A E1021A PR	VcnTS PR	<.0001
VcnTS E1015A E1021A PR	VcnTS SF	<.0001
VcnTS E1015A E1021A SF	VcnTS SF	<.0001
VcnTS E1015A E1021A PR	VcnTS E1015A E1021A SF	0.1236
VcnTS PR	VcnTS SF	0.967
VcnTS PR	VcnTS E1015A E1021A SF	<.0001

Supplementary Table 17:

Delta FRET Eff (of Line Scan Representative FAs) Statistical Significance (one-way ANOVA, post-hoc Steel Dwass test)		
**Level**	**- Level**	***p*-value**
VcnTS PR	VcnTS E1015A E1021A SF	0.2827
VcnTS E1015A E1021A PR	VcnTS E1015A E1021A SF	0.8195
VcnTS E1015A E1021A PR	VcnTS PR	0.116
VcnTS PR	VcnTS SF	0.0114
VcnTS E1015A E1021A PR	VcnTS SF	<.0001
VcnTS E1015A E1021A SF	VcnTS SF	<.0001

Supplementary Table 18:

FA Area All Cells Statistical Significance (one-way ANOVA, post-hoc Steel Dwass test)		
**Level**	**- Level**	***p*-value**
VcnTS_SF	VcnTS PR	<.0001
VcnTS E1015A E1021A SF	VcnTS PR	<.0001
VcnTS E1015A E1021A SF	VcnTS E1015A E1021A PR	<.0001
VcnTS E1015A E1021A PR	VcnTS PR	0.3509
VcnTS E1015A E1021A SF	VcnTS SF	0.0222
VcnTS E1015A E1021A PR	VcnTS SF	<.0001

Supplementary Table 19:

FRET Eff Mean Statistical Significance (one-way ANOVA, post-hoc Steel Dwass test)		
**Level**	**- Level**	***p*-value**
VcnTS E1015A E1021A PR	VcnTS SF	<.0001
VcnTS E1015A E1012A SF	VcnTS SF	<.0001
VcnTS E1015A E1021A PR	VcnTS PR	<.0001
VcnTS E1015A E1012A SF	VcnTS PR	<.0001
VcnTS SF	VcnTS PR	0.8622
VcnTS E1015A E1012A SF	VcnTS E1015A E1021A PR	0.3968

Supplementary Table 20:

Spatial Variation Index Statistical Significance (one-way ANOVA, post-hoc Steel Dwass test)		
**Level**	**- Level**	***p*-value**
VcnTS SF	VcnTS PR	0.0002
VcnTS E1015A E1021A SF	VcnTS E1015A E1021A PR	0.233
VcnTS E1015A E1021A SF	VcnTS PR	0.9392
VcnTS E1015A E1021A PR	VcnTS PR	0.7112
VcnTS E1015A E1021A SF	VcnTS SF	<.0001
VcnTS E1015A E1021A PR	VcnTS SF	<.0001

Supplementary Table 21: **List of salt-bridge and non-bonded interactions between F-actin and Vt in the $F_{pointed}$ direction.**

Salt-bridge interactions	Non-bonded interactions
R1039 (Vt) – E93 (F-actin)	A1043 (Vt) – Y91 (F-actin)
E908 (Vt) – R95 (F-actin)	I977 (Vt) – Y91 (F-actin)
E1042 (Vt) – R95 (F-actin)	N980 (Vt) – H87 (F-actin)
R1049 (Vt) – E99 (F-actin)	T990 (Vt) – L349 (F-actin)
E1036 (Vt) – K50 (F-actin)	I997 (Vt) – I345 (F-actin)
	T993 (Vt) – I345 (F-actin)
	M1022 (Vt) – S145 (F-actin)
	M1022 (Vt) – I330 (F-actin)
	M1022 (Vt) – R147 (F-actin)
	L998 (Vt) – A144 (F-actin)
	L998 (Vt) – S145 (F-actin)
	Q994 (Vt) – G146 (F-actin)
	Q994 (Vt) – Y143 (F-actin)
	Q994 (Vt) – L142 (F-actin)
	T1000 (Vt) – D25 (F-actin)

Supplementary Table 22: **System Setup for Umbrella Sampling Molecular Dynamics Simulations.**

Property	Value
Simulation Box Dimensions	200.3 Å x 120.3 Å x 120.3 Å
Total Number of Atoms	285,629
Total Number of Water Molecules	90,419
Salt Concentration	150 mM NaCl
Temperature	310 K
Pressure	1.0 bar
Integration Time Step	2 fs
Force Field	CHARMM36
Periodic Boundary Conditions	Yes
Electrostatic Treatment	Particle Mesh Ewald (PME)
Cutoff Radii	14 Å
Ensemble	NPT
Pulling Simulation Time	10 ns
Pull Rate	0.01 nm/ps
Simulation Package	GROMACS-2018
