# Supplementary material for: Molecular basis and cellular functions of vinculin-actin directional catch bonding
Source: Nat Commun. 2023 Dec 14;14:8300. doi: 10.1038/s41467-023-43779-x (PMC10721916; doi:10.1038/s41467-023-43779-x)
Supplement: Supplementary file 3 — Description of additional supplementary files [file 41467_2023_43779_MOESM3_ESM.pdf]

Description of additional supplementary files

Supplementary Movie 1: Constantforce pulling of Vt by harmonically restraining the position of F-actin in F-actin:Vt complex at F= 125 pN in the Fpointed direction.

Supplementary Movie 2: Constantforce pulling of Vt by harmonically restraining the position of F-actin in F-actin:Vt complex at F= 125 pN in the Fbarbed direction.

Supplementary Movie 3: Constantforce pulling of Vt by harmonically restraining the position of F-actin in F-actin:Vt complex at F= 125 pN in the Fnormal direction.

Supplementary Movie 4: Constantforce pulling of Vt by harmonically restraining the position of F-actin in F-actin:Vt complex at F= 128 pN in the Fpointed direction.

Supplementary Movie 5: Constantforce pulling of Vt by harmonically restraining the position of F-actin in F-actin:Vt complex at F= 128 pN in the Fbarbed direction.

Supplementary Movie 6: Constantforce pulling of Vt by harmonically restraining the position of F-actin in F-actin:Vt complex at F= 128 pN in the Fnormal direction.

Supplementary Dataset 1: Coordinates of the force-loaded state of Vt:F-actin complex in the $F_{pointed}$ direction.

Supplementary Dataset 2: Coordinates of the unloaded state of Vt:F-actin complex in the $F_{pointed}$ direction.
