# Supplementary material for: Molecular basis and cellular functions of vinculin-actin directional catch bonding
Source: Nat Commun. 2023 Dec 14;14:8300. doi: 10.1038/s41467-023-43779-x (PMC10721916; doi:10.1038/s41467-023-43779-x)
Supplement: Supplementary file 12 — Reporting Summary [file 41467_2023_43779_MOESM12_ESM.pdf]

nature portfolio

Corresponding author(s): Brenton D. Hoffman and Sharon L. Campbell

Last updated by author(s): Oct 6, 2023

# Reporting Summary

Nature Portfolio wishes to improve the reproducibility of the work that we publish. This form provides structure for consistency and transparency in reporting. For further information on Nature Portfolio policies, see our Editorial Policies and the Editorial Policy Checklist.

## Statistics

For all statistical analyses, confirm that the following items are present in the figure legend, table legend, main text, or Methods section.

| n/a | Confirmed | |
|---|---|---|
| ☐ | ☒ | The exact sample size (*n*) for each experimental group/condition, given as a discrete number and unit of measurement |
| ☐ | ☒ | A statement on whether measurements were taken from distinct samples or whether the same sample was measured repeatedly |
| ☐ | ☒ | The statistical test(s) used AND whether they are one- or two-sided<br>*Only common tests should be described solely by name; describe more complex techniques in the Methods section.* |
| ☐ | ☒ | A description of all covariates tested |
| ☐ | ☒ | A description of any assumptions or corrections, such as tests of normality and adjustment for multiple comparisons |
| ☐ | ☒ | A full description of the statistical parameters including central tendency (e.g. means) or other basic estimates (e.g. regression coefficient) AND variation (e.g. standard deviation) or associated estimates of uncertainty (e.g. confidence intervals) |
| ☐ | ☒ | For null hypothesis testing, the test statistic (e.g. *F*, *t*, *r*) with confidence intervals, effect sizes, degrees of freedom and *P* value noted<br>*Give P values as exact values whenever suitable.* |
| ☒ | ☐ | For Bayesian analysis, information on the choice of priors and Markov chain Monte Carlo settings |
| ☒ | ☐ | For hierarchical and complex designs, identification of the appropriate level for tests and full reporting of outcomes |
| ☒ | ☐ | Estimates of effect sizes (e.g. Cohen's *d*, Pearson's *r*), indicating how they were calculated |

*Our web collection on statistics for biologists contains articles on many of the points above.*

## Software and code

Policy information about availability of computer code

| | |
|---|---|
| Data collection | Image acquisition was controlled by Metamorph Advances Software (Olympus). The πDMD software employed for constant force pulling simulations can be requested from Molecules in Action, LLC (http://www.moleculesinaction.com). GROMACS 2018 package can be downloaded from http://www.gromacs.org/. Eris server can be accessed at https://dokhlab.med.psu.edu/eris/login.php. |
| Data analysis | Publicly available, custom written MATLAB scripts on Github (Hoffmanlab) were used to perform image preprocessing ('FRET-Preprocessing' repository), FRET analysis and FA segmentation ('FRET-Processing' repository), and actin segmentation ('Voronoi and Actin Quantification' repository). Simulation trajectories were analyzed using GROMACS 2018 package (http://www.gromacs.org/). Representative 3D figures of Actin-Vt complex were visualized using PyMOL- 2.5.4 (https://pymol.org/2/#download) and Visual Molecular Dynamics (VMD) (https://www.ks.uiuc.edu/Research/vmd/). Computational data plots were generated using Grace program (https://plasma-gate.weizmann.ac.il/Grace/) and Circos tool (version 0.69-8) (http://circos.ca/). The densitometry analysis for actin and lipid co-sedimentation studies were performed using ImageJ software. Representative figures of actin and lipid co-sedimentation analysis were generated using Student Version of Origin software (OriginLab Corporation, MA-01060, USA). |

For manuscripts utilizing custom algorithms or software that are central to the research but not yet described in published literature, software must be made available to editors and reviewers. We strongly encourage code deposition in a community repository (e.g. GitHub). See the Nature Portfolio guidelines for submitting code & software for further information.

## Data

Policy information about availability of data

All manuscripts must include a data availability statement. This statement should provide the following information, where applicable:

- Accession codes, unique identifiers, or web links for publicly available datasets
- A description of any restrictions on data availability
- For clinical datasets or third party data, please ensure that the statement adheres to our policy

The datasets generated during and/or analyzed during the current study are available from the corresponding authors on reasonable request, and plot source data with statistical test p-values have been provided with this manuscript.

## Human research participants

Policy information about studies involving human research participants and Sex and Gender in Research.

| | |
|---|---|
| Reporting on sex and gender | N/A |
| Population characteristics | N/A |
| Recruitment | N/A |
| Ethics oversight | N/A |

Note that full information on the approval of the study protocol must also be provided in the manuscript.

# Field-specific reporting

Please select the one below that is the best fit for your research. If you are not sure, read the appropriate sections before making your selection.

☒ Life sciences ☐ Behavioural & social sciences ☐ Ecological, evolutionary & environmental sciences

For a reference copy of the document with all sections, see nature.com/documents/nr-reporting-summary-flat.pdf

# Life sciences study design

All studies must disclose on these points even when the disclosure is negative.

| | |
|---|---|
| Sample size | Sample sizes were determined based on criteria established in Gates et al., Cytometry A 2018, which demonstrated that differences in 1-2% FRET efficiency can be resolved at large sample sizes (>30 cells). For three experimental days for each Vcn sensor experiment, at least 20-30 independent cell measurements were acquired. |
| Data exclusions | For all TSMod, VcnTS, and VcnCS constructs, only cells with an average acceptor intensity within a pre-specified range within optimal detector range were analyzed. This range was set to [1000 40000] for mTFP1-Venus-based sensors, resulting in exclusion of <10% of cells. Additionally, for VcnTS and VcnCS constructs, cells that were not fully spread were also excluded from analysis. For FA analysis, detected FAs below 8 pixels in size were discarded. These exclusion criteria have been established in LaCroix et al., eLife 2018. |
| Replication | Cellular experiments to determine FA size, tension, conformation, force-sensitive dynamics, actin organization and migration were each repeated for at least three separate days, with cells at different passage numbers. Attempts at replication were successful. |
| Randomization | Cells expressing VcnTS, VcnCS or VcnV constructs were chosen at random for imaging within a dish. |
| Blinding | Blinding was not used in this study. Unbiased analyses were performed using automated image analysis to automatically apply the same analysis conditions to all analyzed images. |

# Reporting for specific materials, systems and methods

We require information from authors about some types of materials, experimental systems and methods used in many studies. Here, indicate whether each material, system or method listed is relevant to your study. If you are not sure if a list item applies to your research, read the appropriate section before selecting a response.

## Materials & experimental systems

| n/a | Involved in the study |
| --- | --- |
| ☐ | ☒ Antibodies |
| ☐ | ☒ Eukaryotic cell lines |
| ☒ | ☐ Palaeontology and archaeology |
| ☒ | ☐ Animals and other organisms |
| ☒ | ☐ Clinical data |
| ☒ | ☐ Dual use research of concern |

## Methods

| n/a | Involved in the study |
| --- | --- |
| ☒ | ☐ ChIP-seq |
| ☒ | ☐ Flow cytometry |
| ☒ | ☐ MRI-based neuroimaging |

# Antibodies

Antibodies used

Anti-GFP primary polyclonal antibody (Abcam, ab290) at a 1:5000 dilution;
GAPDH primary polyclonal antibody (Santa Cruz, Dallas, TX, sc25778) at a 1:3000 dilution

Validation

From Abcam Site (https://www.abcam.com/products/primary-antibodies/gfp-antibody-ab290.html): On Western blot the antibody detects the GFP fraction from cell extracts expressing recombinant GFP fusion proteins and has also been shown to be useful on mouse sections fixed with formalin. In Immunocytochemistry, the antibody gives a very good signal on recombinant YES-GFP chimeras expressed in COS cells (McCabe et al. 1999).
From Santa Cruz website (https://datasheets.scbt.com/sc-25778.pdf): Validation of antibody confirmed via Western blot analysis of GAPDH expression in non-transfected 293T, human GAPDH transfected 293T, and Hep G2 whole cell lysates.

# Eukaryotic cell lines

Policy information about cell lines and Sex and Gender in Research

Cell line source(s)

Cell line: Vinculin Null Mouse Embryonic Fibroblast (Vcn-/- MEF)
Source: Ben Fabry lab. Vcn-/- MEFs were derived from littermate embryos of mice carrying a vinculin null allele that lacks vinculin exon 3 (previously used: https://www.ncbi.nlm.nih.gov/pmc/articles/PMC5954296/)

Authentication

Western blot

Mycoplasma contamination

Negative for mycoplasma

Commonly misidentified lines
(See ICLAC register)

None



March 2021
